# Expansion microscopy of banked brain tissue

**DOI:** 10.17879/freeneuropathology-2026-9593

**Published:** 2026-06-29

**Authors:** Andrew T. McKenzie, Alicia Keberle, Andria Slaughter, Macy Garrood, Ons M'Saad, Jonathan Gulcicek, Ouzéna Bouadi, John F. Crary, Kurt Farrell

**Affiliations:** 1 Apex Neuroscience, Salem, USA; 2 panluminate Inc., New Haven, USA; 3 Friedman Brain Institute, Departments of Pathology, Neuroscience, and Artificial Intelligence & Human Health, Icahn School of Medicine at Mount Sinai, New York, USA; 4 Neuropathology Brain Bank & Research Core and Ronald M. Loeb Center for Alzheimer's Disease, Icahn School of Medicine at Mount Sinai, New York, New York, USA

**Keywords:** Expansion microscopy, Brain banking, Postmortem changes, Neurofilament, γ-protocadherin, Ultrastructural quality

## Abstract

Expansion microscopy (ExM) physically enlarges biological specimens to enable ultrastructural imaging with conventional fluorescence microscopes. However, its performance in postmortem human brain tissue is unclear. Here, we evaluated how a previously established ExM protocol performs on cortical tissue from eight banked brains with postmortem intervals ranging from 40 minutes to 91 hours, and compared these data with electron microscopy (EM) on a subset of matched samples. Both techniques revealed similar patterns of ultrastructural features, including irregular, rounded, unstained spaces as postmortem artifacts that increased with longer postmortem intervals. While EM provided superior resolution for synaptic details, ExM enabled more high-throughput volumetric imaging of neural circuits. ExM also enabled molecular annotation of ultrastructure through immunofluorescence, as demonstrated by SMI-312 neurofilament and γ-protocadherin labeling. Our findings show that ExM can visualize aspects of ultrastructure in routinely banked brain tissue. While EM provides better resolution for fine synaptic detail, ExM offers a complementary approach that combines nanoscale imaging with molecular specificity and more accessible high-throughput volumetric capabilities.

## Abbreviations

**AIZ** - Ambiguous interstitial zone, **EM** - Electron microscopy, **ExM** - Expansion microscopy, **FIB-SEM** - Focused ion beam scanning electron microscopy, **NBF** - Neutral buffered formalin, **NCMIR** - National Center for Microscopy and Imaging Research, **NHS** - N-hydroxysuccinimide, **PMI** - Postmortem interval, **PSD95** - Postsynaptic density protein 95, **SMI** - Sternberger Monoclonals Incorporated, **TEM** - Transmission electron microscopy

## Introduction

The ultrastructural characterization of brain tissue is a unique window for understanding both normal neural architecture and pathological changes in neurobiological disorders. The ability to visualize subcellular structures such as synapses, organelles, and cytoskeletal elements provides insights into brain function and dysfunction that cannot be obtained through conventional light microscopy (1). Electron microscopy (EM) has long been the primary option for ultrastructural imaging, offering resolution down to the nanometer scale and enabling visualization of fine cellular details such as synapses and thin cellular processes. However, EM has several limitations, including a high cost of the microscopes, relatively slow imaging of large volumes, and challenges in performing molecular annotation of the observed structures. Expansion microscopy (ExM) has emerged as a promising option that can physically expand tissue samples 4- to 20-fold, enabling super-resolution imaging using a conventional fluorescence microscope (2). This technique offers several potential advantages compared to EM, including having a lower cost for imaging larger tissue volumes and being more easily compatible with immunostaining.

Brain banks worldwide collectively hold tens of thousands of postmortem human specimens, representing an enormous potential resource for ultrastructural studies (5,6). Although ExM has shown substantial promise in various neuroscience applications, its performance in postmortem brain tissue, and especially banked human brain tissue, requires further evaluation. Previous studies have successfully applied ExM to surgically resected human brain tissue and postmortem samples, demonstrating the feasibility of the technique in human specimens (7). These studies have revealed important biological findings, such as the nature of myelin-axon interface vulnerabilities in Alzheimer disease (9). However, to the best of our knowledge, characterizing the ultrastructural preservation quality of postmortem brain samples has not been a primary focus of extant studies using ExM. This is important because the vast majority of human brain tissue available for research comes from brain banks with variable agonal states, postmortem intervals (PMIs), and storage conditions. Furthermore, to the best of our knowledge, no comparisons between ExM and EM have been performed on matched tissue samples from banked brains. As a result, there is a critical need to evaluate how ExM compares to the gold standard of EM in banked human brain tissue.

Our prior EM study of postmortem human brain tissue identified ambiguous interstitial zones (AIZs), which are non-membrane-bound, unstained regions of uncertain origin. These became more prevalent at longer postmortem intervals (PMIs), and seemed to particularly obscure unmyelinated axons (11). We hypothesized that these AIZs could be caused in part by visualization artifacts rather than true structural loss. This is because EM primarily visualizes lipids through osmium binding, which may be especially sensitive to postmortem degradation. In contrast, ExM visualizes protein structures that may be more stable postmortem. We reasoned that one way to test the hypothesis that some protein structures might still be present in at least a subset of AIZs would be to compare ExM and EM images from matched banked brain samples.

In this manuscript, we present results from a pilot study comparing ExM and EM on matched tissue samples from eight banked brains with PMIs ranging from 40 minutes to 91 hours. Our overall goal was to characterize the performance of a previously published ExM approach (3) in this tissue context. We had three primary objectives. First, to characterize the ultrastructural preservation quality achievable with ExM in a sample of postmortem tissue with varying PMIs and fixation durations. Second, to evaluate the relative strengths and limitations of ExM and EM for visualizing key ultrastructural features such as synapses and axonal processes. And third, to measure the molecular annotation capabilities of ExM on banked tissue via immunolabeling of selected antigens.

## Methods

### Brain banking procedures

Anatomical whole-body donations were performed by a partner whole-body donation organization operating under Oregon Health Authority regulations. Additionally, two deceased canines were donated through our canine brain bank program, following euthanasia by a licensed veterinarian, with signed owner consent for research use (12). The Apex Neuroscience Brain and Tissue Bank operates under an exemption determination issued by the Pearl Institutional Review Board (IRB) after the submission of our protocols for review.

### Tissue samples and preparation

Brain banking methods were performed as previously described (11). To summarize, tissue samples for both ExM and EM were obtained from the frontal pole of both human and canine brains with varying postmortem intervals (PMIs). All brains underwent perfusion fixation with 10 % neutral buffered formalin (NBF), with variable efficacy (13), followed by immediate immersion fixation and fluid preservation in 10 % NBF at 4 °C prior to shipment and processing.

### Expansion microscopy

Brain tissue expansion was performed by panluminate Inc. through a contracted service, using a prototype reagent kit in development, which builds on a previously published method that performs expansion of the tissue with retention of proteins followed by bulk staining of the proteins in the tissue (thus, "pan"-staining) (3). Briefly, fixed tissue was sectioned at 70 μm thickness and incubated in a solution containing acrylamide and formaldehyde for protein anchoring. Sections were then embedded in an expansion gel solution and placed in Milli-Q water to achieve gel expansion. Gels were subsequently re-embedded, and this process was repeated iteratively, yielding linear expansion factors of approximately 15- to 17.7-fold across samples. A detailed description of the underlying expansion method is provided in (3), while the use of the prototype reagent kit is available through a service provided by panluminate Inc. The names "PF" and "NPF" in the publicly available images on Zenodo, an open-access research data repository, refer to additional post-fixation treatment conditions that were tested to optimize tissue anchoring to the hydrogel, which were found not to significantly affect the structural features analyzed in this study.

The tissue was pan-stained with Atto 488 NHS ester (Sigma-Aldrich, #41698) in a single labeling step to label proteins non-specifically. For the samples that were not expanded, they were pan-stained with Atto 488 NHS ester separately. Neurofilaments were visualized using the SMI-311 antibody (BioLegend, #837801; 1:200) and SMI‑312 antibody (BioLegend, #837904; 1:200). SMI-311 staining was unsuccessful. Detection of the SMI‑312 antibody was performed using a goat anti-mouse IgG (H+L) CF640R conjugate (Biotium, #20175). PSD95 was visualized using the rabbit anti-PSD95 antibody (Cell Signaling, #3450; 1:250). γ-protocadherin was visualized using the mouse anti-pan-γ-protocadherin antibody (NeuroMab, #75-185; 1:250). For signal amplification of γ-protocadherin, we used the FRACTAL (Fluorescent Signal Amplification via Cyclic Staining of Target Molecules) method, specifically following the previously published "simple" FRACTAL protocol and using its associated buffers (MAXblock Blocking Medium #15252, MAXbind Staining Medium #15251, and MAXwash Washing Medium #15254) (14). The mouse anti-pan-γ-protocadherin primary antibody (NeuroMab, #75-185; 1:250) was applied first and detected with a goat anti-mouse StarRed secondary antibody (Abberior, #52283; 1:250). The signal was then amplified by alternating an unconjugated mouse anti-goat bridging antibody (Invitrogen, #31107; 1:250) with the goat anti-mouse StarRed secondary. This cycle was repeated for four rounds, with washes between steps.

Images were acquired on an Andor BC43 spinning disk confocal microscope equipped with a CFI Apochromat LWD Lambda S 40×/1.15 water immersion objective. Pan-stained proteins (Atto 488 NHS ester) were imaged using a 488 nm excitation wavelength. Neurofilaments labeled with goat anti-mouse IgG (H+L) CF640R were imaged using a 638 nm excitation wavelength. γ-protocadherin labeled with goat anti-mouse StarRed was imaged using a 630 nm excitation wavelength. PSD95 labeled with CF568 was imaged using a 562 nm excitation wavelength.

Two types of confocal imaging were performed: (1) overview images consisting of 2D 10×10 stitches of large tissue areas to assess general structural preservation, homogeneity, and signal intensity; and (2) stack images comprising 3D acquisitions of single or multiple fields of view with 0.2 μm z-steps to evaluate structural continuity in the z-direction. The resulting images were adjusted for brightness in order to aid in visual clarity.

Depth coloring of z-stacks was performed using the Z-stack Depth Color Code plugin (version 0.0.2), which assigns colors from a lookup table based on the z-position of each optical section (15). This visualization technique enables the three-dimensional organization of neural structures to be represented in 2D projections, with color indicating the relative depth within the tissue volume.

### Electron microscopy

The electron microscopy images analyzed in this study are a subset of those reported in (11), used here for comparison with expansion microscopy data from matched tissue samples. Tissue samples were processed for electron microscopy as previously described in (11). Briefly, tissue was post-fixed in 2 % paraformaldehyde and 2.5 % glutaraldehyde in 0.1 M sodium cacodylate buffer, then processed using an adapted NCMIR (National Center for Microscopy and Imaging Research) protocol for enhanced contrast (16). This included sequential treatments with tannic acid, reduced osmium, thiocarbohydrazide, osmium, and uranyl acetate at room temperature, followed by lead aspartate staining at 60 °C. Samples were dehydrated through graded ethanol, infiltrated with Embed 812 epoxy resin (EMS), and polymerized for 72 hours at 60 °C. Ultrathin sections (70 nm) were cut using a Leica UC7 ultramicrotome and collected on nickel slot grids. Imaging was performed on cortical layers II/III of each sample. Images were acquired on a HT7500 transmission electron microscope (Hitachi High-Technologies, Tokyo, Japan) using an AMT NanoSprint12 12-megapixel CMOS TEM Camera System, with minimal contrast adjustments applied during acquisition. One of the cortical samples prepared as described above for EM was also imaged by focused ion beam scanning electron microscopy (FIB-SEM); specifically, multi-angle plasma FIB milling. Image stacks were acquired with 24 nm section thickness and in-plane pixel sizes of 8 nm and 16 nm. The volume used for neurite tracing was acquired at 8 × 8 × 24 nm voxel size and consisted of 215 sections.

### Neurite tracing

To measure the traceability of neurites in our imaged tissue volumes, we used a sampling approach based on prior methods for evaluating circuit reconstruction fidelity in volume EM data (17,18). For each imaging modality (FIB-SEM and ExM), 50 synapses were randomly identified within the image volume from human donor 7. For each synapse, the presynaptic and postsynaptic neurites were independently traced through the z-stack, yielding 100 neurite traces per modality. Notably, the annotations of the two neurites as the pre- and postsynaptic sides are our best estimates, but may be inaccurate, and this does not affect our tracing analysis. Tracing proceeded from the synaptic contact in both directions through serial optical sections (ExM) or serial FIB-SEM slices (EM) until the neurite either reached the boundary of the imaged volume, merged with a parent dendrite or axon, or became untraceable due to signal loss, inability to disambiguate, AIZs, or other imaging limitations. The traceable distance for each neurite was recorded as a percentage of the total stack depth and also converted to an absolute distance (in μm) based on the known depth of the imaged sample. Tracing was performed in Imaris Viewer for ExM data and in Fiji for EM data.

## Results

### Pan-staining of banked brain samples

We performed expansion microscopy on cortical tissue samples from six human and two canine brains with PMIs ranging from 40 minutes to 91 hours (**Table 1**). All samples achieved robust expansion factors between 15.0- and 17.7-fold.

**Table:** **1:** Characteristics of brain donors included in this study.

**Donor ID**	**Age (Years)**	**Sex**	**PMI**	**Reported cause of death**	**Duration of fixation at 4 °C**	**Expansion factor**
177**	14	Male	40 min	Euthanasia (Donated canine)	3 months	17.2
65**	17	Female	1.5 h	Euthanasia (Donated canine)	9.5 months	15.0
7	78	Male	4.25 h	Cancer*	12.5 months	16.7
182	62	Male	5 h	Pancreatic cancer	2 months	15.6
247	79	Male	14 h	Bladder cancer*	1 month	16.2
34	88	Male	20 h	Cancer*	15 months	16.1
37	70	Male	72 h	Myocardial infarction	15 months	17.7
59	41	Female	91 h	Leukemia	14 months	17.5

Duration of fixation refers to the amount of time in 10 % neutral buffered formalin at 4 °C. Expansion factor refers to the degree of linear expansion of the sample. *: This donor utilized medical aid in dying (MAID) for end-of-life care. **: Canine brain. PMI: Postmortem interval.

**Figure 1: Representative images of expansion microscopy images of pan-stained samples taken from the frontal cortex:**
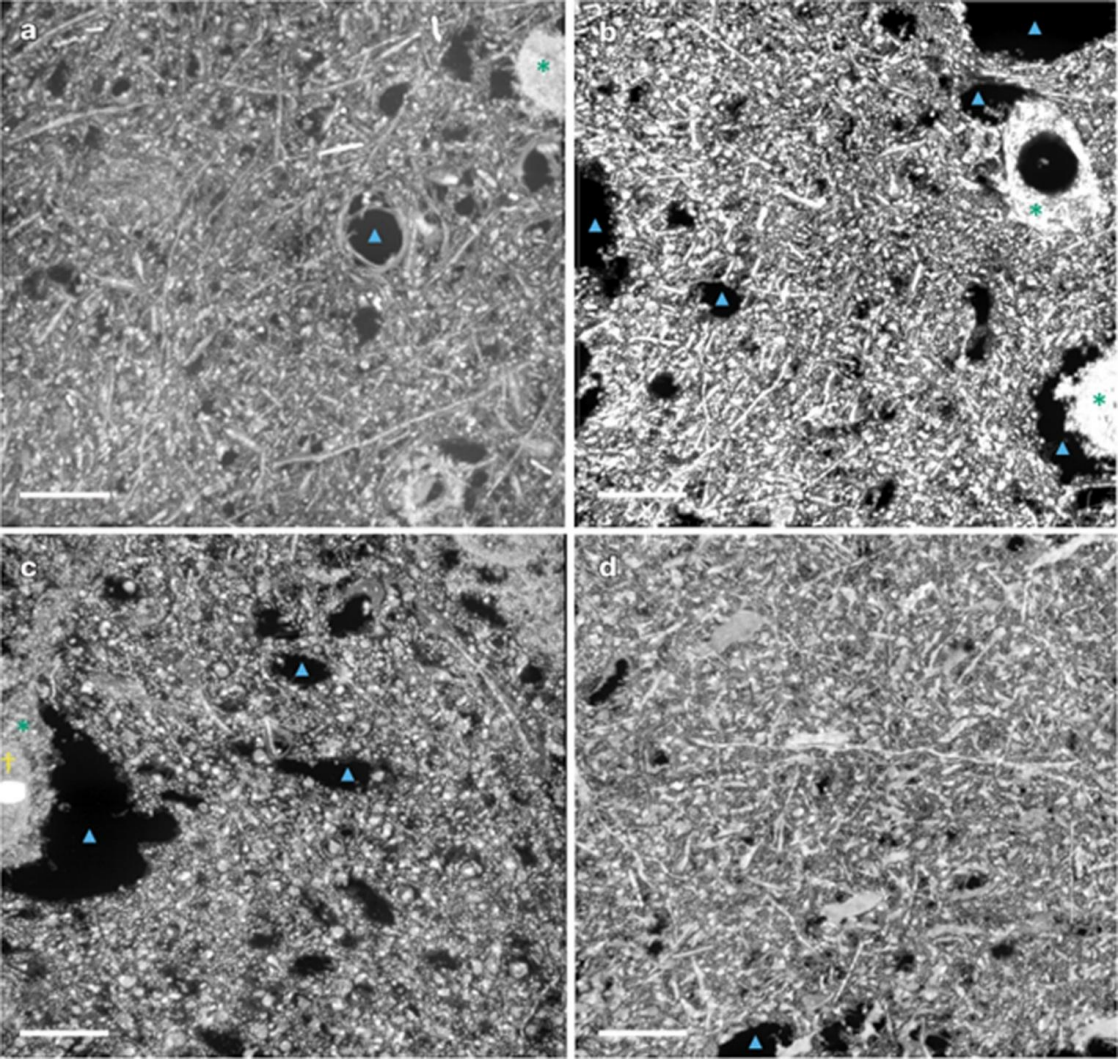
The tissue was pan-stained with Atto 488 NHS ester, which non-specifically labels protein and thereby renders cell bodies, processes, and neuropil as fluorescent (bright) structures. Green asterisks indicate structures consistent with cell bodies. The yellow dagger indicates a structure consistent with a nucleus. The large, irregular, rounded black regions are non-fluorescent unstained spaces, a subset of which are annotated with blue arrowheads. We interpret these unstained spaces as primarily corresponding to swollen astrocyte processes resulting from postmortem changes, on the basis of previous literature (19). Donor numbers and PMIs: 34, 20 hours (**a**); 59, 91 hours (**b**); 37, 72 hours (**c**); and 247, 14 hours (**d**). All scale bars 100 μm post-expansion; pre-expansion: 6.2 μm (**a**), 5.7 μm (**b**), 5.6 μm (**c**), 6.2 μm (**d**).

Pan-protein staining with Atto 488 NHS ester allowed visualization of tissue architecture across all samples, though with varying degrees of postmortem changes (**Figure 1**; **Supplementary File 1**). In all samples, cell bodies appeared as distinct circular or ovoid structures with the shape of the nucleus generally preserved, surrounded by a dense meshwork of neural processes. We note that without immunostaining for specific protein markers, we cannot definitively determine the identity of the structures observed in the pan-stained images.

**Figure 2: Pre- and moderate-expansion images show that unstained spaces are not an artifact of expansion:**
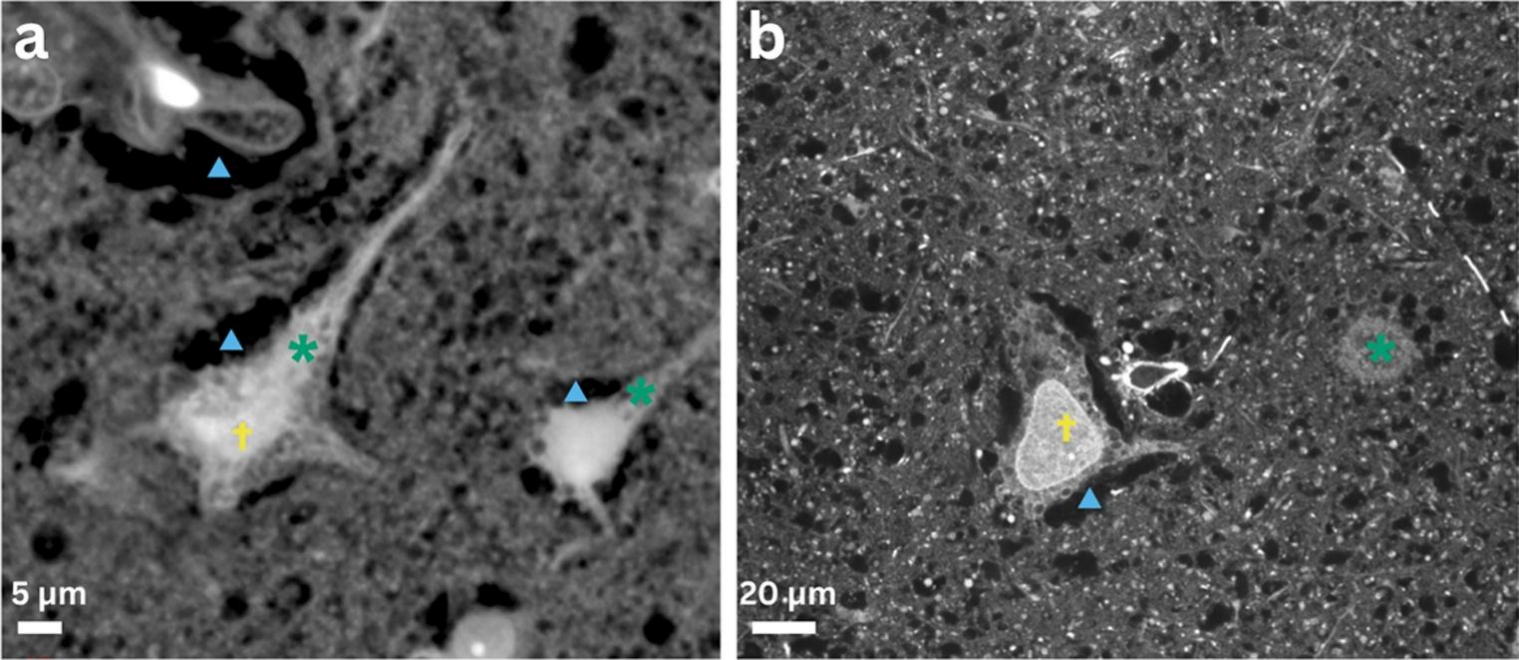
Pre-expansion (**a**) and approximately 4x-expanded (**b**) samples were each stained with Atto 488 NHS ester, which labels protein non-specifically. Red asterisks indicate structures identified as cell bodies and light green daggers indicate structures identified as nuclei. The large, irregular, rounded black regions are non-fluorescent unstained spaces, which we interpret as primarily corresponding to swollen astrocyte processes resulting from postmortem changes. Note that the unstained spaces are retained as the sample expands and become more easily recognized as the resolution increases. The fields of view shown are in an approximately equivalent area of tissue for comparison. Human donor 7, PMI of 4.25 hours. Scale bars: 5 μm (**a**) and 20 μm (5.0 μm pre-expansion) (**b**).

A consistent finding across all samples was the presence of dark, non-fluorescent, irregular, rounded unstained spaces throughout the expanded tissue. We note that, unlike with electron microscopy, it is not possible to easily distinguish whether these areas are membrane-bound or not, and therefore we cannot determine whether they would be classified as AIZs (11). These unstained spaces were particularly concentrated in pericellular or perivascular regions. While present even in samples with shorter PMIs, they became noticeably more abundant and broader in samples with longer PMIs. Pre-expansion control images showed the presence of these unstained spaces as well (**Figure 2**). This finding indicates that they were not artifacts of the expansion process itself, but rather resulted from postmortem changes in the tissue architecture. We expect that these unstained spaces predominantly correspond to the fluid-filled portions of swollen astrocyte processes, because they have a similar location and shape as that type of postmortem change, as previously seen in light microscopy and electron microscopy data (19).

**Figure 3: Representative expansion microscopy images of canine (donor 177;:**
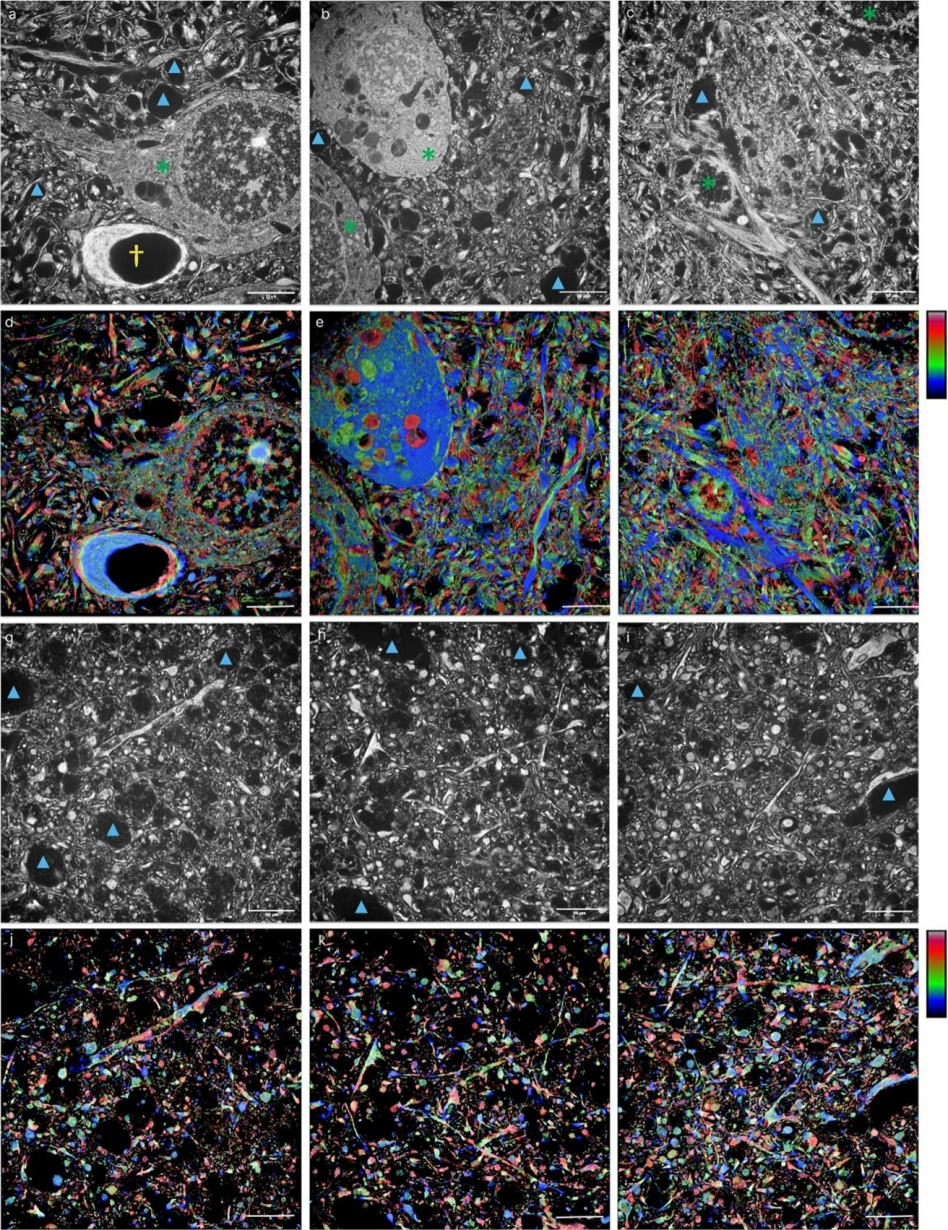
Tissue was pan-protein stained with Atto 488 NHS ester, which non-specifically labels protein. In the grayscale panels (**a–c** and **g–i**), protein-dense structures such as cell bodies, neural processes, and neuropil appear bright, while non-fluorescent unstained spaces appear dark. Green asterisks indicate structures consistent with cell bodies; the yellow dagger indicates a structure consistent with a blood vessel in cross-section, identifiable by a bright, protein-dense rim surrounding a dark lumen; and blue arrowheads indicate a subset of the rounded dark unstained spaces present throughout the tissue. Panels **d–f** and **j–l** show depth-coded renderings of the same regions, with colors indicating relative position within the z-stack (blue: surface, green: middle, red: deep), highlighting the three-dimensional, criss-crossing organization of processes. Scale bars: All 50 μm post-expansion; pre-expansion: 2.9 μm (**a–f**), 3.2 μm (**g–l**). Stack depths: post-expansion 10 μm (**d**, **f**, **j–l**) or 20 μm (**e**); pre-expansion 0.58 μm (**d**, **f**), 0.64 μm (**j–l**), or 1.16 μm (**e**).

On further examination of ExM images, we found that expected cellular structures, such as cellular processes, blood vessels, and nucleoli, could be clearly visualized on ExM images (**Figure 3**). Several fine cellular processes remained resolvable throughout the expanded tissue. Depth coloring of image stacks highlighted the 3D organization of these processes, allowing the crisscross nature of the neuropil easier to appreciate (**Figure 3**). However, because the depth-coloring method we used is partially lossy, we found that it was less effective for visualizing 3D structures in samples with longer PMIs, resulting in more empty (i.e., non-fluorescent) space.

**Figure 4: Representative expansion microscopy images of a cell with a nucleus that appears to show detailed structural features:**
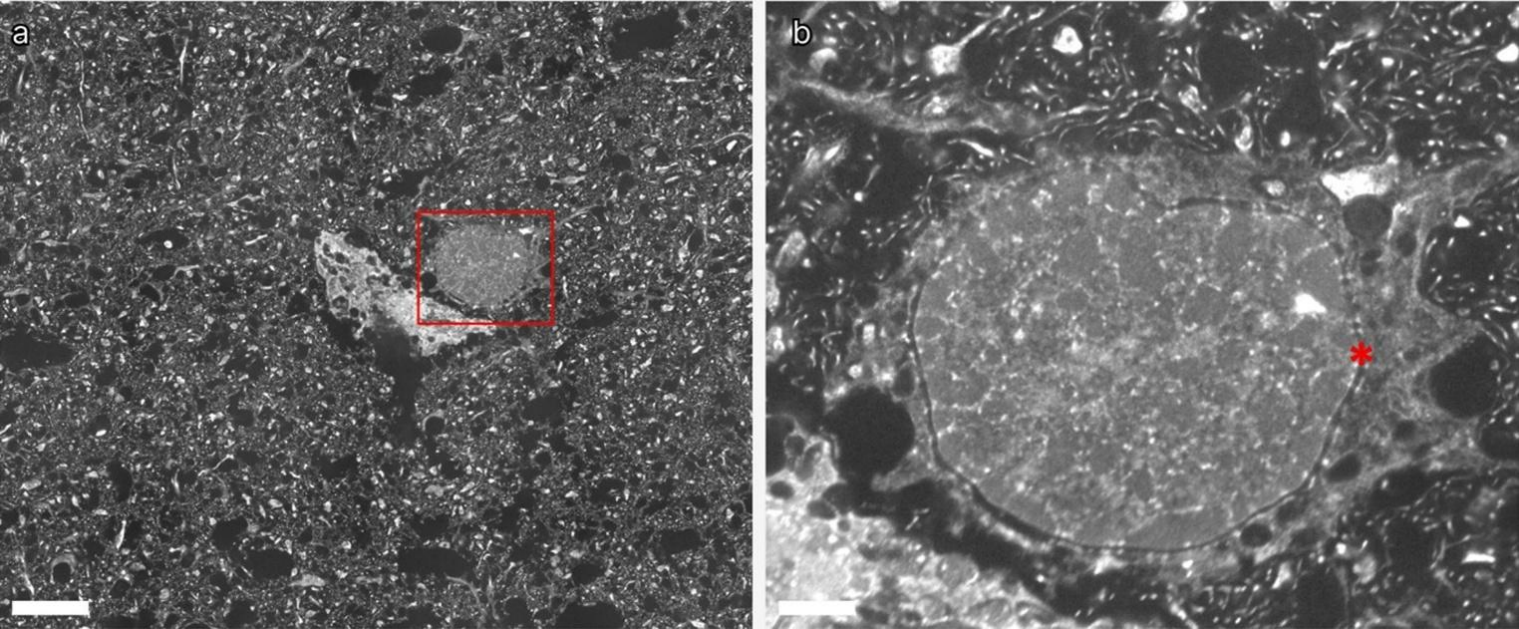
Cortical tissue from donor 247 (PMI 14 hours) was pan-protein stained with Atto 488 NHS ester, which non-specifically labels protein. (**a**) is a lower magnification image containing a red box that is magnified in (**b**). Below the red asterisk in (**b**) is a possible nuclear pore complex. Scale bars: (**a**) 100 μm post-expansion (6.2 μm pre-expansion); (**b**) 20 μm post-expansion (1.2 μm pre-expansion).

In some samples, especially those with shorter PMIs, we identified features morphologically consistent with organelle structures, such as nuclear pore complexes (**Figure 4**). Pan-expansion microscopy has previously demonstrated the ability to visualize nuclear pore complexes (20). However, without immunolabeling, we cannot definitively confirm the identity of these structures in our samples.

### Comparison to electron microscopy

**Figure 5: Representative images allow a comparison of expansion microscopy and electron microscopy images:**
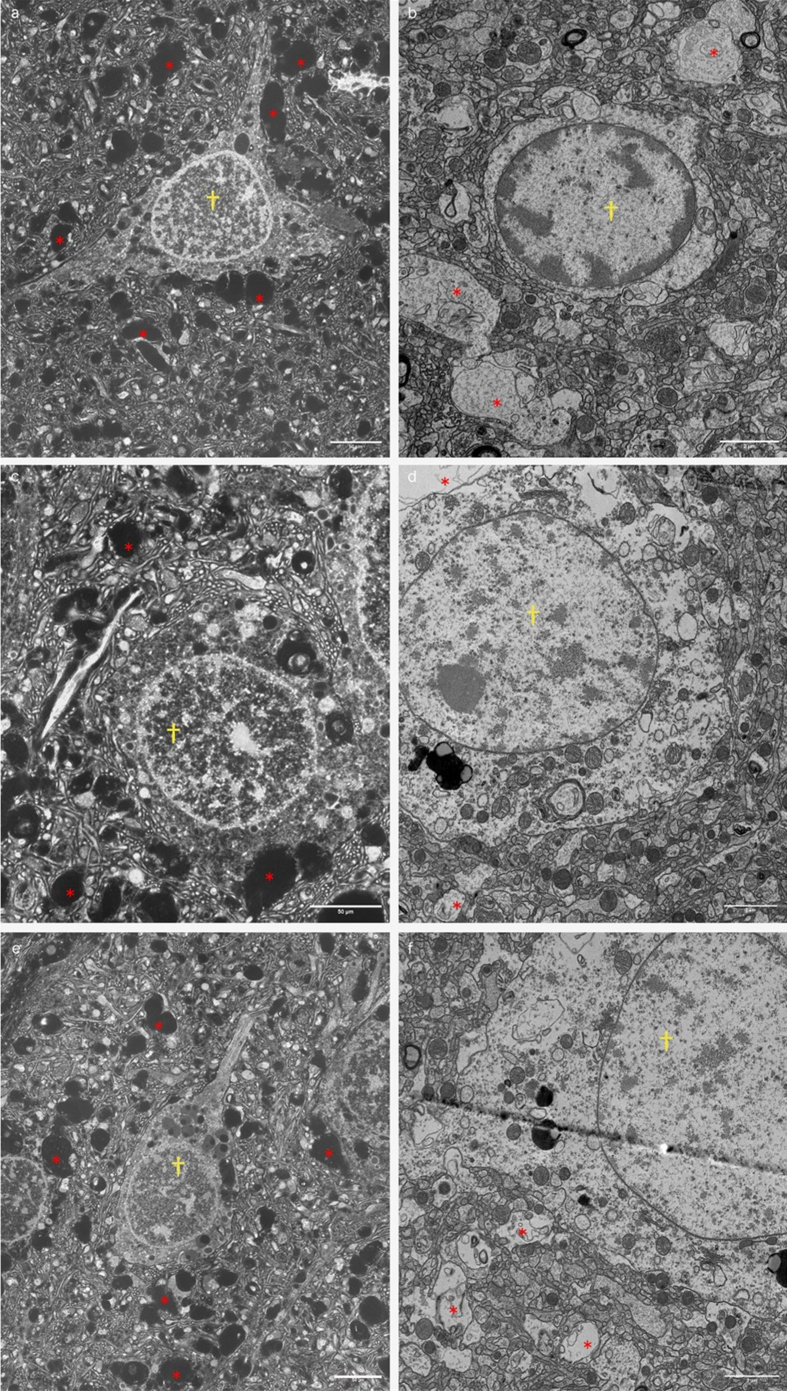
Expansion microscopy images left (**a**, **c**, **e**), and electron microscopy images, right (**b**, **d**, **f**), of samples from human donor number 7 (**a–b**) and canine donor number 65 (**c–f**). The red asterisks indicate unstained spaces in both expansion microscopy and electron microscopy images. The yellow daggers indicate structures consistent with cell nuclei. Scale bars: for ExM panels 50 μm post-expansion (**a**, **c**, **e**) and 3.0 μm (**a**) or 3.3 μm (**c**, **e**) pre-expansion; 2 μm for EM panels (**b**, **d**, **f**).

A comparison of ExM and EM images from matched tissue samples demonstrated that both imaging techniques show similar cellular architecture (**Figure 5**). Additionally, the frequent presence of unstained spaces in images from matched samples using both modalities corroborates that these are postmortem biological changes rather than technique-specific artifacts.

**Figure 6: Comparison of synapse visualization in canine (donor 65,:**
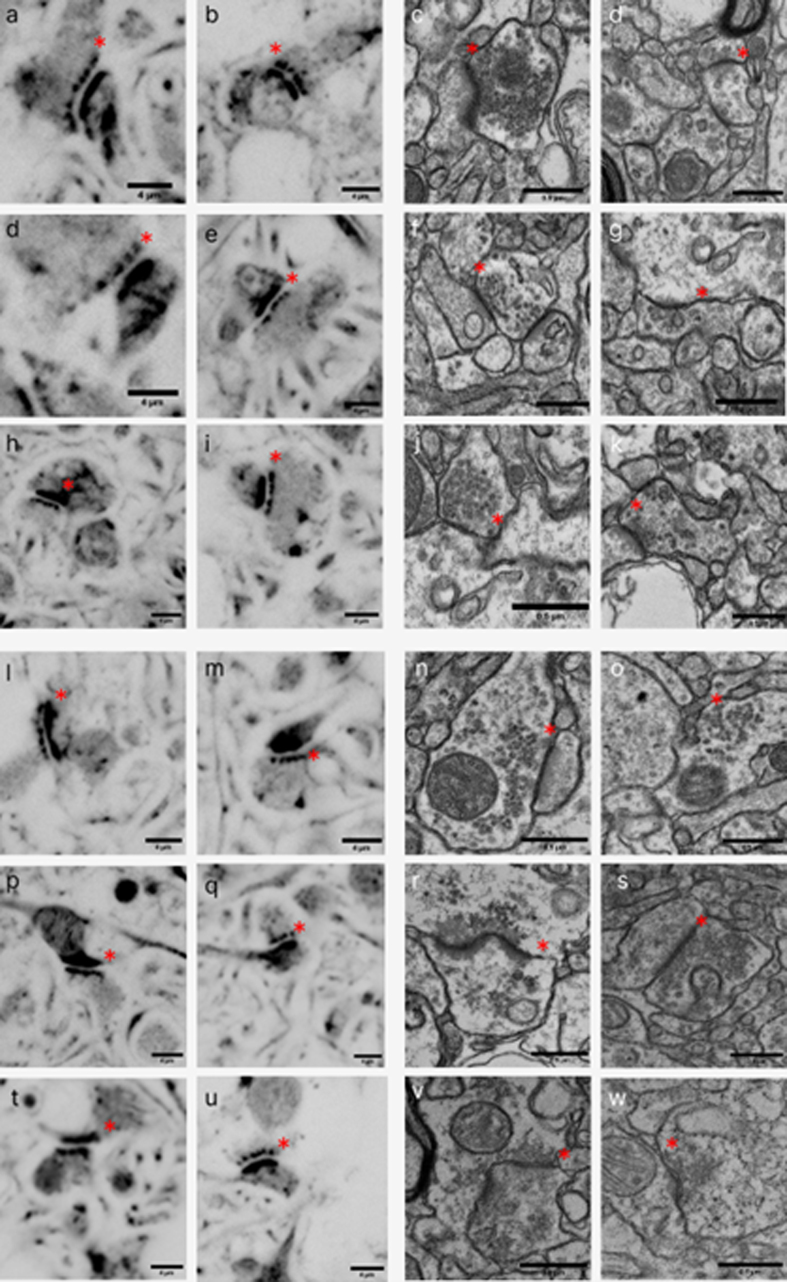
Tissue prepared for expansion microscopy was pan-stained with Atto 488 NHS ester, which non-specifically labels proteins. For expansion microscopy, the colors are inverted. Red asterisks indicate the location of a synapse. Scale bars: for ExM, all 4 μm post-expansion and 0.27 μm (donor 65) or 0.24 μm (donor 7) pre-expansion; all 0.5 μm for EM.

Synapses were well visualized in all of our samples, frequently with well-delineated presynaptic and postsynaptic densities flanking the synaptic cleft (**Figure 6**). This corroborates previous reports that synapses are relatively resilient to postmortem changes (19). In some cases, we could discern clusters in the ExM images of what appear to be synaptic vesicles at presynaptic terminals, although this would require antibody staining for more definitive characterization. However, in the absence of immunolabeling, the fine ultrastructural details within synapses, such as the morphology of synaptic vesicles and perisynaptic mitochondria, were somewhat better resolved using EM, consistent with previous data (21).

In order to measure the traceability of neurites in banked brain tissue, we performed volumetric imaging using both ExM and FIB-SEM on tissue from human donor 7 (PMI: 4.25 hours). For each imaging modality, we randomly sampled 50 synapses and traced both the corresponding presynaptic and postsynaptic neurites as far as possible through the image stack, yielding 100 neurite traces per modality. In the ExM data, 1 % (1/100) of neurites could be traced throughout the entire volume, compared to 6 % (6/100) in the FIB-SEM data (**Supplementary Data Files 2 and 3**). On average, neurites identified in ExM were traced through 14.9 % of the image stack (corresponding to 0.89 μm pre-expansion/14.9 μm post-expansion of the 5.98 μm pre-expansion/100 μm post-expansion depth), compared to 21.4 % for FIB-SEM (corresponding to 1.10 μm of the 5.16 μm depth).

**Figure 7: Neurite tracing from a synapse in matched EM and ExM volumes:**
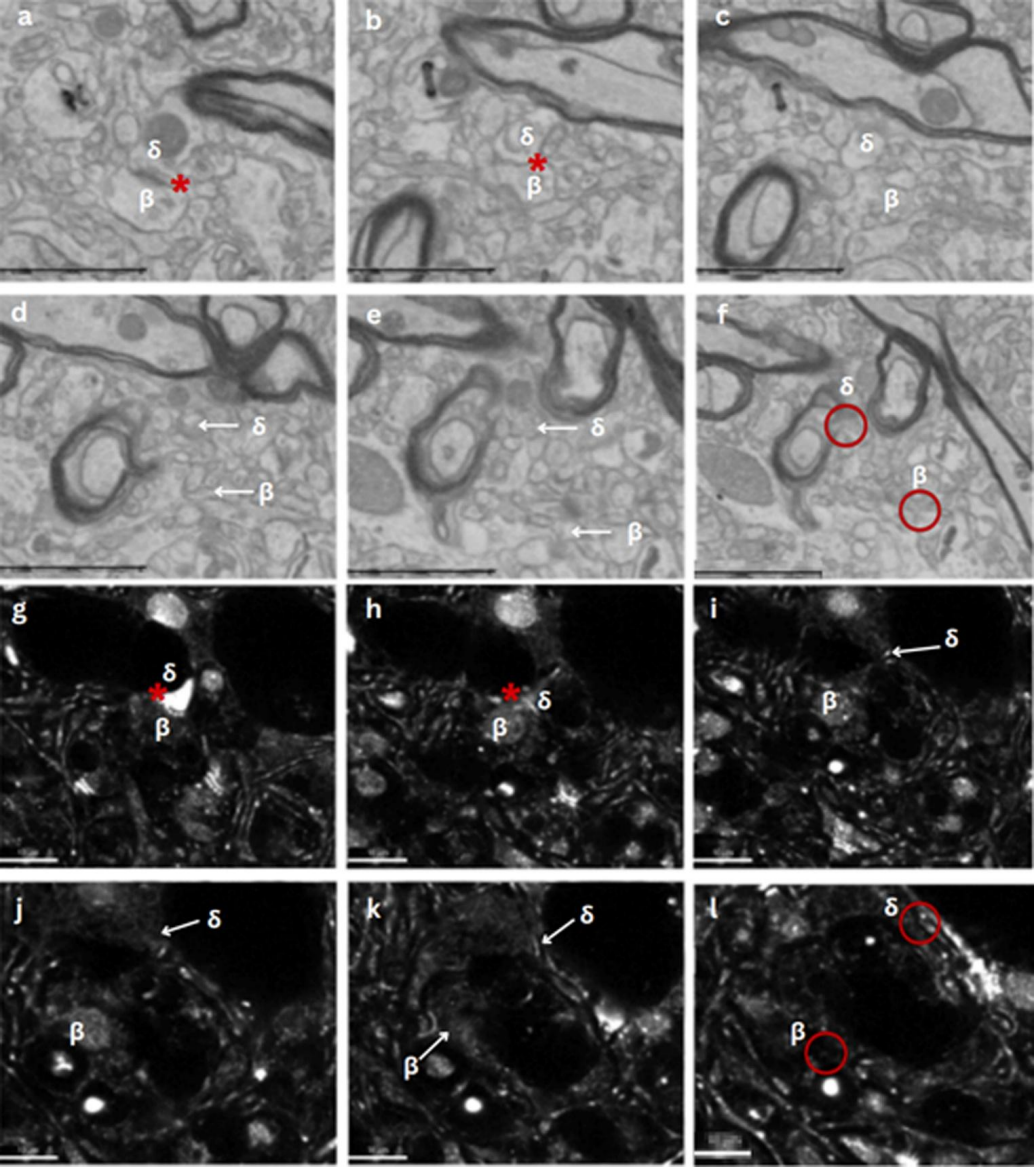
Data are from cortical samples of human donor 7 (PMI: 4.25 hours). (**a–f**) Serial FIB-SEM sections sampled at 10-section intervals, showing the presynaptic (δ) and postsynaptic (β) neurites traced from the synaptic contact (red asterisk). By panel f, both neurites become difficult to unambiguously identify (red circles). (**g–l**) Corresponding ExM optical sections sampled at 10-section intervals. The synapse (red asterisk) and the presynaptic and postsynaptic neurites are similarly traced. As in EM, the neurite identities become ambiguous in deeper sections (red circles in panel **l**). Scale bars: 500 nm (**a–f**); for ExM, 10 μm post-expansion, 0.6 μm pre-expansion (**g–l**).

As previously discussed, in these volumetric datasets, the synapses were typically distinct in both modalities, possibly due to their high concentration of biomolecules at the pre- and postsynaptic densities. On the other hand, their associated neurites more commonly fell below detection thresholds. In the brain, most local synaptic connections are wired by thin, unmyelinated axons and dendrites (22). These thin neurites may be more susceptible to postmortem degradation and produce weaker signals after both pan-staining for ExM and heavy metal staining for EM. This finding contrasts with our previous serial section EM data from canine cortex (donor 65, PMI: 1.5 hours), in which neurites originating from synapses could be successfully traced across multiple sections, though that analysis was less comprehensive due to a smaller volume of imaging data (11). The shorter PMI of this canine sample may have contributed to the improved traceability in that dataset (Figure 7).

### Molecular staining of expanded tissue

**Figure 8: SMI-312 neurofilament immunolabeling of expanded cortical tissue:**
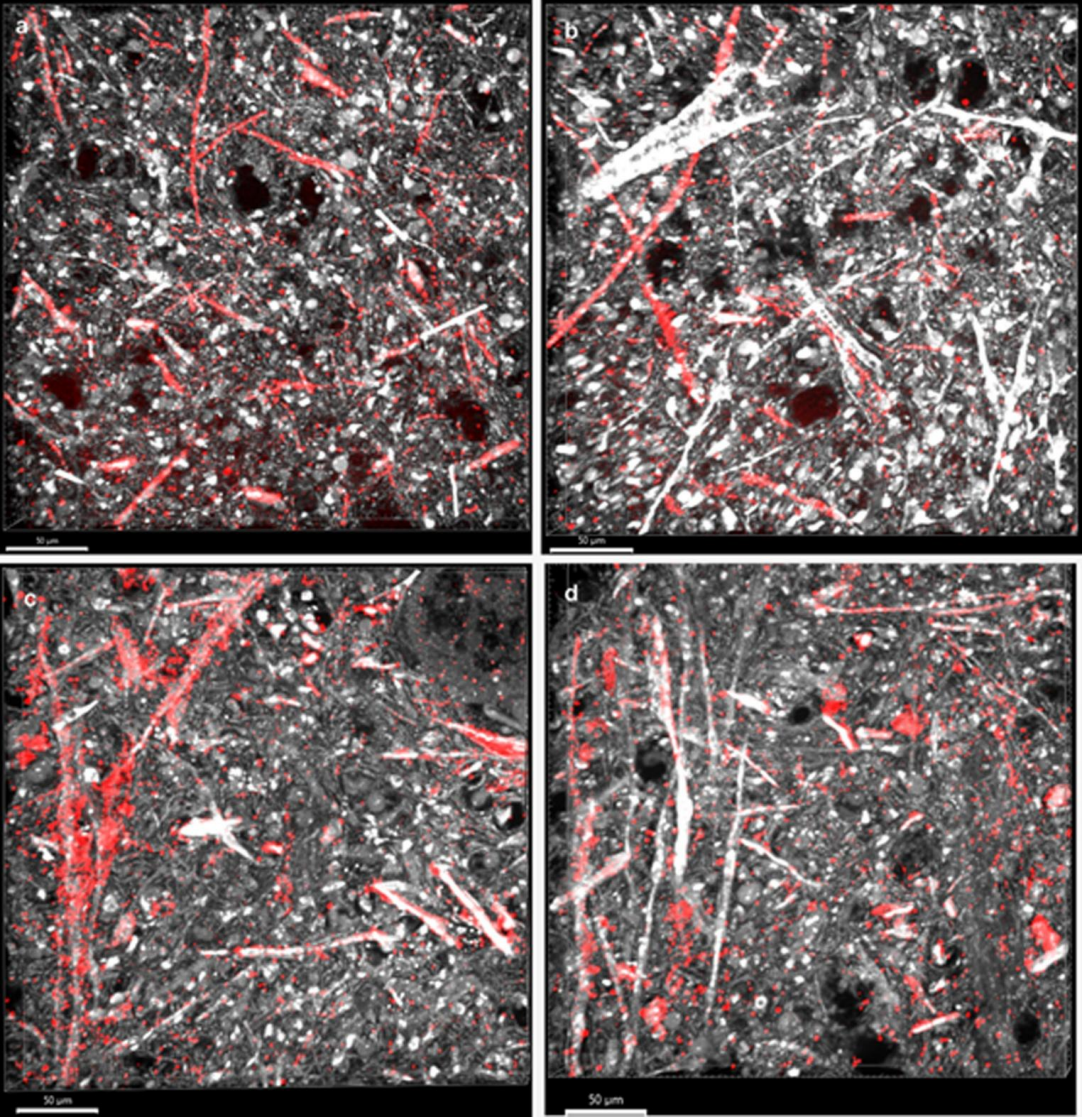
Pan-protein stain (white/gray) and SMI-312 immunofluorescence (red) in human donor 7 (**a**, **b**) and canine donor 65 (**c**, **d**). SMI-312 labels a subset of processes consistent with axons, while many additional processes are visible only on the pan-stain. Scale bars: All 50 μm post-expansion; pre-expansion 3.0 μm (**a**, **b**) or 3.3 μm (**c**, **d**).

To evaluate the molecular annotation capabilities of ExM, we performed immunofluorescence labeling with SMI-312, a pan-axonal neurofilament marker, on expanded cortical tissue from human donor 7 and canine donor 65 (**Figure 8**). We had initially hypothesized that a cytoskeletal-specific stain might reveal additional processes not detected by the pan-stain, but the pan-stain consistently labeled more processes than SMI-312. The SMI-312-positive structures were morphologically consistent with axons based on their elongated, relatively uniform caliber, while many pan-stain-visible processes lacked immunoreactivity. The unlabeled processes likely include dendrites and glial processes (1) that would not be expected to express the neurofilament epitopes targeted by SMI-312, although postmortem degradation or epitope dilution following expansion may also have reduced the labeling of some neurofilament-expressing axons.

**Figure 9: γ-protocadherin and PSD95 co-labeling at moderate and full expansion:**
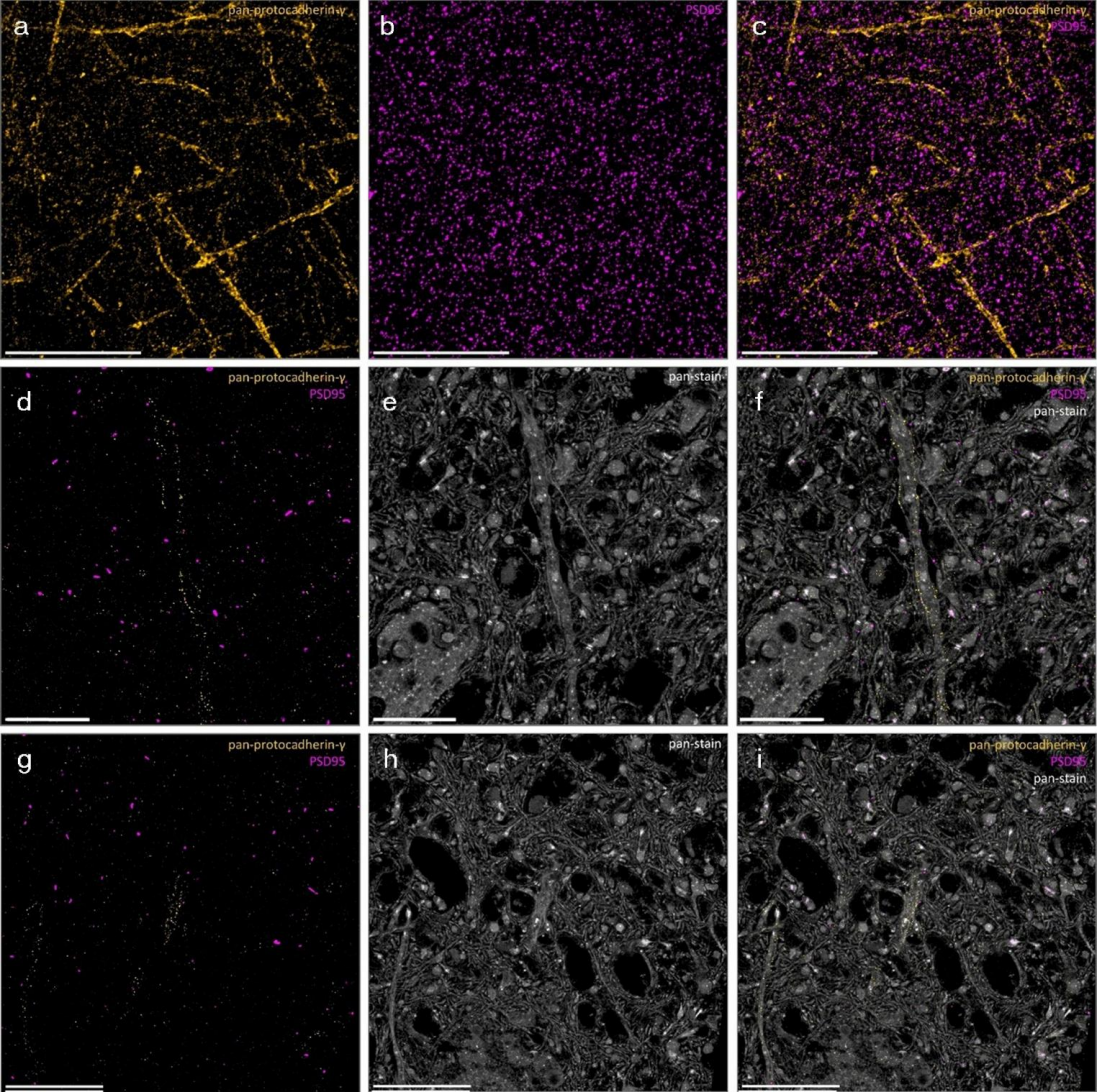
Pan-γ-protocadherin (yellow) and PSD95 (purple) at ∼4x expansion (**a-c**). Pan-γ-protocadherin and PSD95 (yellow/purple), pan-stain (gray), and merged images at ∼16x expansion from two fields of view (**d-f**, **g-i**). Both signals are substantially diminished at full expansion. Human donor 7 (PMI: 4.25 hours). Scale bars: all 50 μm post-expansion; pre-expansion 12.5 μm (∼4x, **a-c**) or 3.0 μm (∼16x, **d-i**).

We next labeled γ-protocadherin and PSD95 to test the feasibility of multiplexed molecular annotation on expanded human brain bank tissue. At moderate expansion of approximately 4x, pan-γ-protocadherin immunofluorescence revealed labeled processes with a morphology consistent with neurites, while PSD95 produced a punctate staining pattern largely consistent with postsynaptic densities (**Figure 9**). PSD95 was also found in structures consistent with neuronal cell bodies, consistent with some previous observations (23), which may be a pool of synthesized protein in the soma. However, at 16x expansion, both immunostaining signals were substantially diminished (**Figure 9**).

**Figure 10: Signal amplification rescues γ-protocadherin immunolabeling at full expansion:**
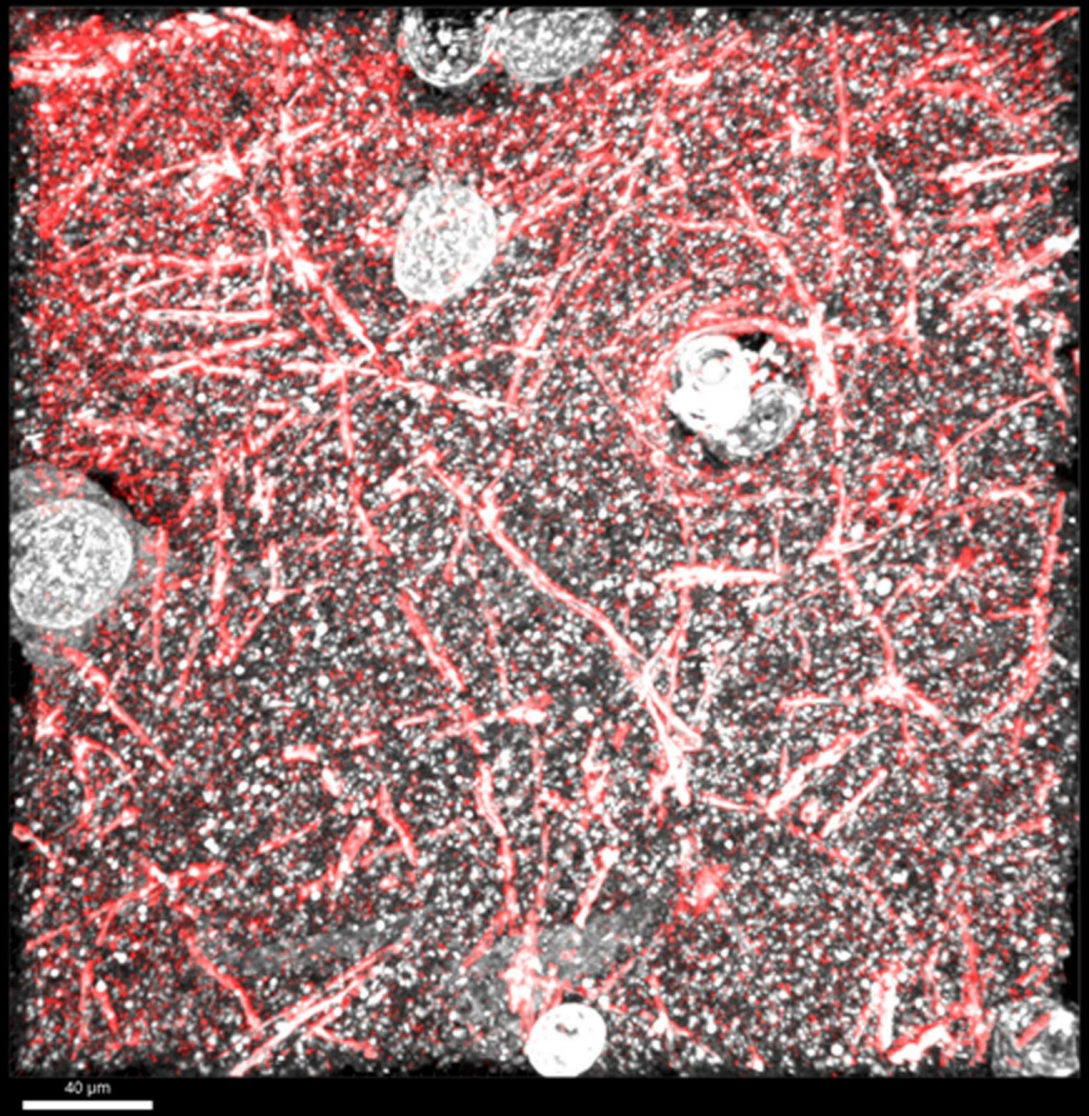
Pan-γ-protocadherin immunofluorescence (red) and pan-protein stain (gray) of fully expanded (∼16x) cortical tissue from human donor 7 (PMI: 4.25 hours) after FRACTAL signal amplification. γ-protocadherin positive processes are visible throughout the neuropil. Scale bar: 40 μm post-expansion, 2.4 μm pre-expansion.

To address the signal loss at higher expansion factors, we applied a FRACTAL signal amplification protocol to the γ-protocadherin immunolabeling (14). This approach uses iterative rounds of secondary antibody staining to build up the signal on the primary target. Signal amplification led to a substantial increase in signal at both moderate and full expansion, compared to unamplified controls imaged with identical acquisition settings. With amplification, γ-protocadherin labeling was clearly detectable in neurite-like processes at full (∼16x) expansion (**Figure 10**). In the merged image, processes positive for γ-protocadherin could be seen throughout the neuropil alongside pan-stain-labeled structures. Although it is only from one sample, this preliminary result suggests that γ-protocadherin is expressed broadly across neurites in the neuropil of elderly human cerebral cortex, rather than being restricted to a small subset.

## Discussion

In this study, we compared ExM and EM on matched cortical tissue samples from eight banked brains with postmortem intervals ranging from 40 minutes to 91 hours. Our findings demonstrate that ExM can successfully visualize ultrastructural features in banked brain tissue, achieving expansion factors of 15.0- to 17.7-fold across all samples regardless of the PMI or the duration of fixation. The ultrastructural features we focused on consisted primarily of nuclear substructures, synapses, neurites, and other cellular processes. Both ExM and EM revealed similar patterns of cellular architecture and postmortem changes, including the presence of unstained spaces that increased with longer PMIs. EM provided superior resolution for fine synaptic details such as vesicle morphology and perisynaptic organelles. On the other hand, ExM offered advantages in volumetric imaging throughput and the ability to perform molecular annotation via immunofluorescence. These results establish ExM as a useful technique that is complementary to EM for studying the ultrastructure of banked brain tissue, particularly when investigators are interested in molecular annotation or high-throughput imaging of larger tissue volumes.

### Postmortem artifacts

One of our motivations for performing this study was to evaluate whether ExM could better characterize the AIZs that we previously characterized in EM images of postmortem brain tissue (11). Since EM provides better relative visualization of lipid membranes as a result of osmium binding, while ExM targets protein structures through fluorescent labeling, we hypothesized that ExM might reveal structures within AIZs that cannot be seen on EM. Our results did not support this hypothesis. Although we could not directly distinguish AIZs from membrane-bound compartments due to the lack of membrane staining in ExM, we found that pan-stained ExM images exhibited similar patterns of unstained spaces as in EM. And molecular labeling for axon cytoskeletal proteins with SMI-312 did not reveal additional neurite structures within the unstained spaces. The convergence of findings across two different imaging modalities, one based primarily on lipid staining and the other on protein fluorescence, suggests that unstained spaces (including AIZs and membrane-bound structures) are most likely caused by true postmortem tissue changes rather than visualization artifacts specific to the EM staining protocol. However, while these regions currently appear to consist of only fluid, we cannot rule out that certain types of diffuse or degraded biomolecules may still be present within these regions at concentrations below our detection thresholds. If so, targeted labeling of specific molecular species with higher-sensitivity detection methods could still potentially reveal a degree of biomolecular content within at least a subset of these unstained spaces that pan-staining and the antibodies used here did not capture.

Our findings may help to contextualize why brain tissue with substantially longer PMIs can appear well-preserved when visualized with conventional light microscopy methods (19,11). At the resolution of standard light microscopy, cellular architecture, including cell bodies, major processes, and general tissue organization, often remains largely intact even at extended PMIs (24,19). This should not necessarily be considered a limitation of light microscopy, as for many research applications, it provides an accurate and sufficient assessment. However, when investigators desire to visualize nanoscale structural details, such as synaptic morphology or the tracing of neurites, EM and ExM reveal a level of structural detail that light microscopy cannot resolve. And in this context, the PMI appears to have a much stronger effect, especially on the visualization of neurites in volumetric data. As a result, our findings corroborate the idea that apparent preservation quality seen on light microscopy should not be considered a strong form of evidence of whether fine ultrastructural features are also intact.

### Neurite traceability

In connectomics, run-length metrics are commonly used to evaluate neurite segmentation algorithms (25,26). These measure the fidelity of reconstruction by quantifying how far along a neurite an algorithm can trace before encountering an error. This is benchmarked against human-generated ground truth. The implicit assumption behind these measures is that the tissue is well enough preserved and the imaging is of sufficient quality so that a skilled human annotator can, in principle, trace the neurites unambiguously.

In our study, we adapted this type of framework to a different purpose, which is evaluating the effect of postmortem tissue changes on neurite traceability (17). Rather than comparing an automated segmentation to a human ground truth, we measured how far a human tracer could follow the neurites from identified synapses before the signal became ambiguous or was lost entirely. This approach builds on our earlier qualitative observations in serial section TEM, where we found that neurite tracing was qualitatively more difficult in samples with a higher burden of AIZs, although we were unable to quantify traceability in that dataset due to the limited imaging depth and a small number of neurites traced (11). In this study, we overcome these limitations by using volumetric imaging (ExM z-stacks and FIB-SEM) and systematically tracing 100 neurites per modality from 50 randomly selected synapses.

We propose that measuring the traceability of neurites from the synapses they originate from may serve as a useful metric for evaluating ultrastructural preservation quality in banked brain tissue. This metric captures the degree to which the synaptic connectivity could be reconstructed from a given set of images, which is the key consideration for any potential connectomic analysis of banked brain tissue, whether it is performed by ExM or EM. However, an important caveat is that neurite traceability is a proxy not only of the initial preservation quality (such as the PMI), but also the resolution and contrast characteristics of the sample preparation and imaging modality. For example, postmortem degradation is expected to reduce the density of stainable biomolecules within thin neurites, potentially rendering structures that are still physically present below the detection threshold of a given imaging method. As a result, the traceability of a sample should be interpreted as a composite metric of both the tissue quality and the capabilities of the imaging method used.

### Molecular annotation

Here, we demonstrate a couple of ways that molecular labeling could be paired with ExM imaging. Our observation that SMI-312 labeled only a subset of cellular processes seen after pan staining is consistent with the known specificity of this antibody for phosphorylated neurofilaments enriched in axons. Many of the unlabeled processes are likely dendrites, while fine astrocyte and other glial processes, which constitute a substantial fraction of cortical neuropil (27), would similarly lack neurofilament immunoreactivity. Additional factors such as postmortem degradation of neurofilament epitopes and dilution of epitope density following expansion may also play a role in decreasing the staining. Disentangling these factors would require co-labeling with dendritic markers or glial markers in future studies. Nonetheless, the potential ability to distinguish axonal from non-axonal processes at ultrastructural resolution would be an advantage of ExM over EM, where such molecular distinctions would require more challenging immunogold labeling or correlative approaches.

Our γ-protocadherin and PSD95 co-labeling results also show the feasibility of multiplexed molecular annotation on expanded human brain bank tissue. If established, protocadherin staining could, in principle, provide a different form of data for tracing neural connectivity patterns in preserved tissue where the morphological tracing results alone are ambiguous. Protocadherins are cell adhesion molecules highly expressed in the nervous system that are present at a subset of synapses and in intracellular compartments within neurites (28,29). They generate high combinatorial diversity through the stochastic choice of promoters – i.e., whereby individual neurons activate different subsets of protocadherin promoters – and this expression pattern is stably maintained in a given neuron over time (30). This allows individual neurons to perform self/non-self recognition along their neurites, which is required for neurite self-avoidance and proper neural circuit assembly. From a technological perspective, the expression of protocadherin isoforms could theoretically provide an endogenous molecular barcode for individual neurons.

Here, we show that γ-protocadherin can be recognized in neurites of a 78-year-old human donor in expanded cortical tissue, suggesting that the spatial distribution of this protein is retained in banked brain tissue. Therefore, if a neurite becomes structurally untraceable due to postmortem degradation, it might in theory be possible to infer its identity if the same protocadherin isoforms could be detected on both sides of a gap. However, actually imaging the tissue in this manner would require substantial advances, most critically the ability to uniquely label individual protocadherin isoforms rather than the pan-γ-protocadherin antibody used here. There would also need to be further characterization of protocadherin copy number and distribution along neurites. Other molecular labels could be used as well to identify ambiguous structures, such as PSD95 if synapses were damaged. This idea of using molecular labeling to better characterize tissue structure after damage (e.g. due to the PMI) is speculative, but it is a theoretical benefit of ExM that might become more relevant in the future.

### Limitations

This study has several limitations. First, our sample size of eight brains is relatively small, limiting the generalizability of our findings. Second, our neurite tracing comparison was performed on a single human donor, which limits the generalizability of the results. This should be thought of as an exploratory analysis of how that type of assessment can be performed. Finally, we did not perform systematic quantification of the density or size of unstained spaces across the visualization methods, instead relying on qualitative assessments.

Future work would benefit from more quantification of imaging results and specifically more quantification of the preservation quality. However, methods for how to do so are still, to the best of our knowledge, not well established. Another direction for future work would be to characterize ExM quality in tissue handled under other common brain banking conditions, such as after preservation via immersion fixation alone, which we did not address here. Relatedly, combining pan-staining with pathology-specific immunolabeling (such as for amyloid-beta or phosphorylated tau) could allow ExM to characterize age-related neurodegenerative changes at ultrastructural resolution, which we did not attempt here.

## Conclusions

Our results show that ExM can visualize ultrastructural features in routinely banked brain tissue, even after fixation durations of over a year. It seems that there are many specimens in brain banks that could be usefully profiled with ExM, without necessarily requiring specialized collection or storage protocols. However, as with EM, we observed that shorter PMIs yield substantially better ultrastructural preservation. This corroborates the conventional wisdom that rapid tissue preservation is important for studies of fine structures beyond what conventional light microscopy can assess. Based on our findings, EM is preferable when characterizing high-resolution synaptic morphology is the primary goal. On the other hand, ExM appears to be better suited for molecular annotation or for performing high-throughput volumetric imaging with widely available fluorescence microscopes. Taken together, we find that the two approaches have different strengths, and that ExM is particularly well suited for combining molecular annotation with nanoscale structural imaging across relatively large tissue volumes.

## Author contributions

A.T.M., J.F.C., and K.F. conceptualized the study. A.K., A.S., M.G., O.M., J.G., and O.B. performed laboratory experiments. A.T.M., A.K., and A.S. performed data analysis. A.T.M. and A.S. wrote the initial draft of the manuscript. All authors reviewed the manuscript and approved the final manuscript.

## Data availability

The ExM data, and the EM data that are new to this paper, can be publicly accessed on Zenodo, available in these three repositories: https://zenodo.org/records/19616438, https://zenodo.org/records/19666865, and https://zenodo.org/records/19669788.

## Declaration of Generative AI Technologies

In the preparation of this manuscript, the authors used Claude (Anthropic) for both programming assistance and to improve the manuscript’s language. All AI tool-assisted content was reviewed and edited by the authors, who take full responsibility for the final publication.

## Conflict of interest statement

Andrew McKenzie, Alicia Keberle, Andria Slaughter, and Macy Garrood are or were employees of Sparks Brain Preservation, a non-profit brain preservation organization. Ons M'Saad, Jonathan Gulcicek, and Ouzéna Bouadi are employees of panluminate Inc.

## Supplementary material

Supplementary Data File 1 (PDF file; 693 KB)

Supplementary Data File 2 (PDF file; 15.429 KB)

Supplementary Data File 3 (PDF file; 7.299 KB)

## Supplementary Material

Supplementary Data File 1 (PDF file; 693 KB)

Supplementary Data File 2 (PDF file; 15.429 KB)

Supplementary Data File 3 (PDF file; 7.299 KB)
