# Supplementary material for: Expansion microscopy of banked brain tissue
Source: Free Neuropathol. 2026 Jun 29;7:15. doi: 10.17879/freeneuropathology-2026-9593 (PMC13344129; doi:10.17879/freeneuropathology-2026-9593)
Supplement: Supplementary file 1 [file freeneuropathol-07-15-9593-s1.pdf]

DOI: https://doi.org/10.17879/freeneuropathology-2026-9593

Supplementary Data File 1

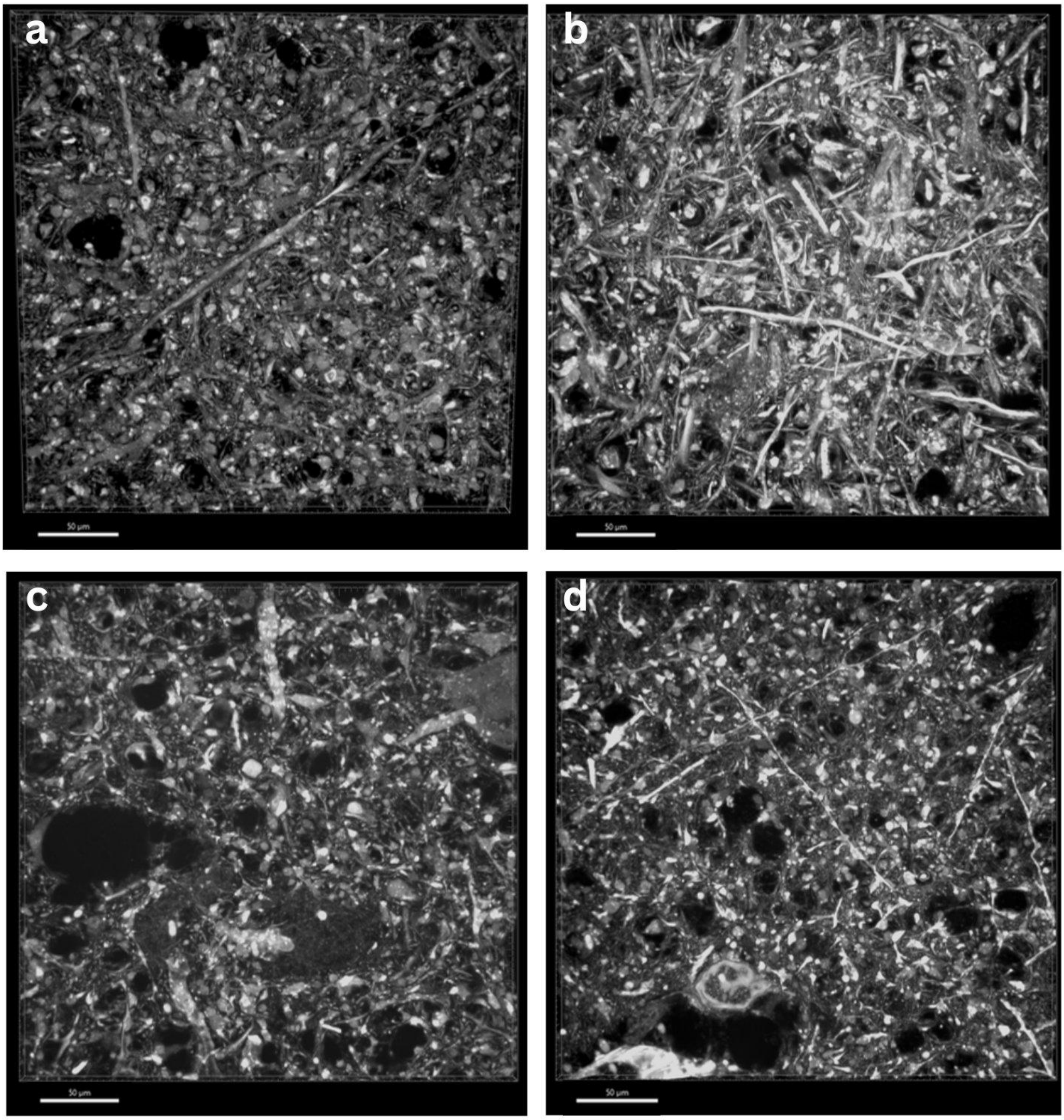

Supplementary Data File 1. Additional representative images of expansion microscopy images of pan-stained samples taken from the frontal cortex.

The tissue was pan-stained with Atto 488 NHS ester, which non-specifically labels protein and thereby renders cell bodies, processes, and neuropil as fluorescent (bright) structures. Donor numbers and PMIs: 65, 1.5 hours (**a**); 177, 40 minutes (**b**); 182, 5 hours (**c**); and 7, 4.25 hours (**d**). All scale bars 50 µm post-expansion; pre-expansion: 3.0 µm (**a**), 2.9 µm (**b**), 3.2 µm (**c**), 3.0 µm (**d**).

Copyright: © 2026 The author(s). This is an open access article distributed under the terms of the Creative Commons Attribution 4.0 International License (https://creativecommons.org/licenses/by/4.0/), which permits unrestricted use, distribution, and reproduction in any medium, provided the original author and source are credited, a link to the Creative Commons license is provided, and any changes are indicated. The Creative Commons Public Domain Dedication waiver (https://creativecommons.org/publicdomain/zero/1.0/) applies to the data made available in this article, unless otherwise stated.
