# Supplementary material for: Expansion microscopy of banked brain tissue
Source: Free Neuropathol. 2026 Jun 29;7:15. doi: 10.17879/freeneuropathology-2026-9593 (PMC13344129; doi:10.17879/freeneuropathology-2026-9593)
Supplement: Supplementary file 2 [file freeneuropathol-07-15-9593-s2.pdf]

Supplementary Data File 2

Synapse #	Image	Ability to trace a	Ability to trace b	% Traced through
1	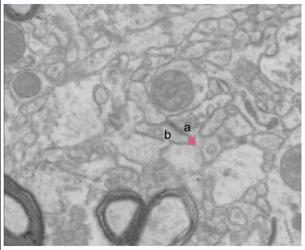	169-189	169-215	21.4
2	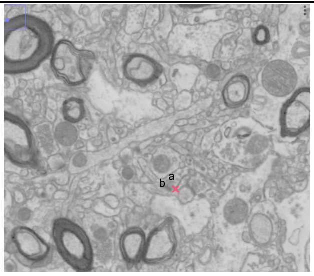	167-200	167-215	22.33
3	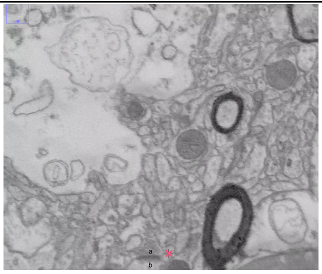	1-18	1-20	9.3
4	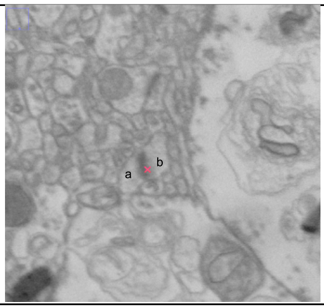	69-125	69-100	26.05
5	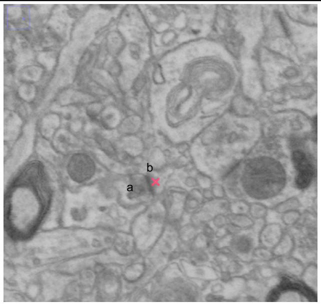	36-44	36-58	10.23
6	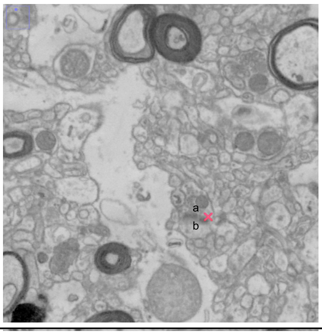	93-110	93-100	7.9

Synapse #	Image	Ability to trace a	Ability to trace b	% Traced through
7	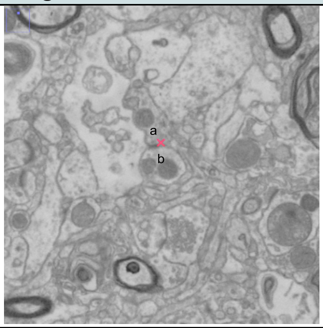	84-110	76-110	15.81
8	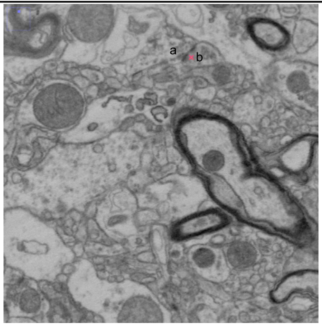	1-25	1-23	11.63
9	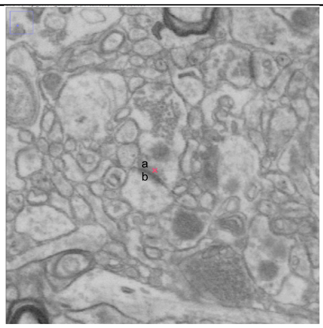	15-30	15-40	11.63
10	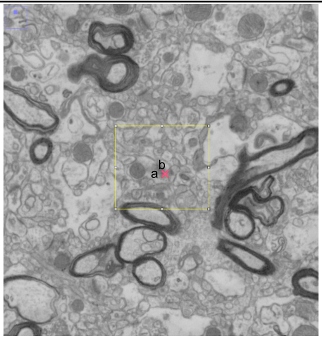	20-65	20-40	20.93
11	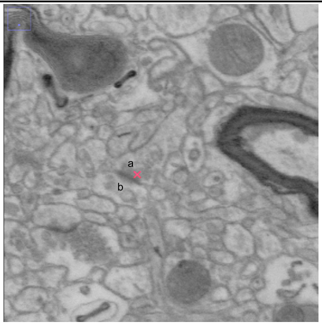	24-62	24-44	17.67

Synapse #	Image	Ability to trace a	Ability to trace b	% Traced through
**12**	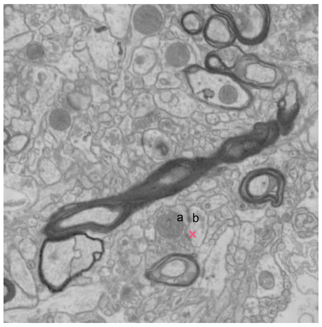	71-105	71-95	15.81
**13**	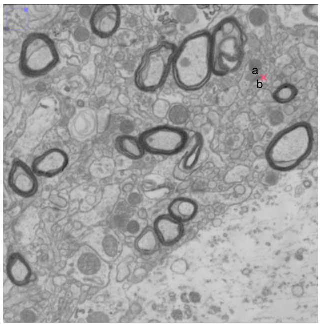	99-115	99-120	9.77
**14**	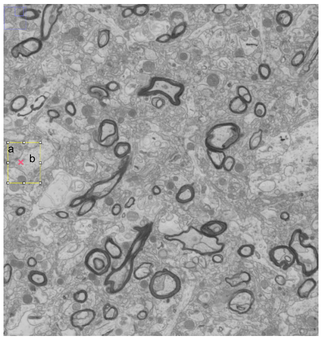	36-79	36-65	20
**15**	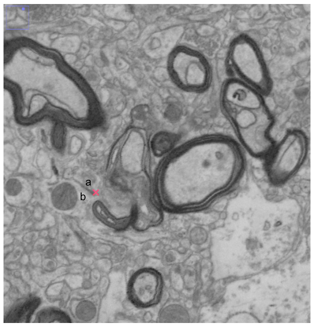	165-215	165-213	23.26
**16**	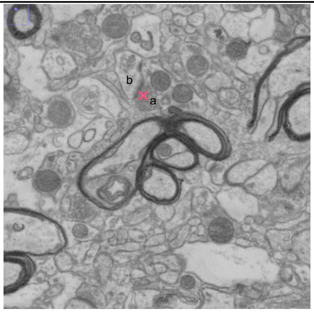	1-40	1-56	26.04

Synapse #	Image	Ability to trace a	Ability to trace b	% Traced through
17	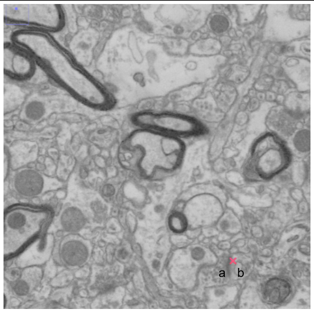	38-78	38-75	18.6
18	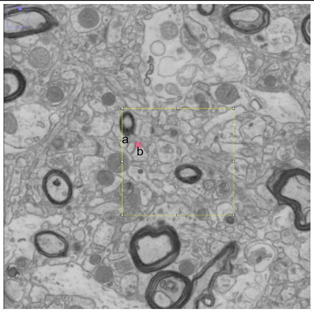	20-88	20-70	31.63
19	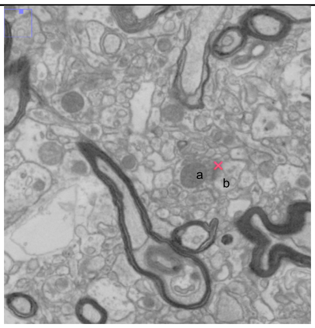	1-33	1-40	18.6
20	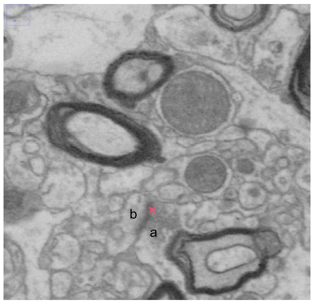	2-27	2-22	11.63
21	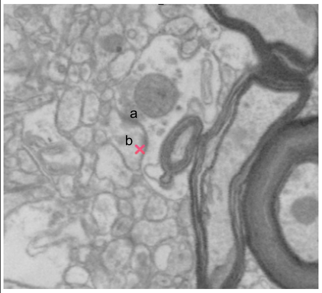	137-215	137-215	36.28

Synapse #	Image	Ability to trace a	Ability to trace b	% Traced through
22	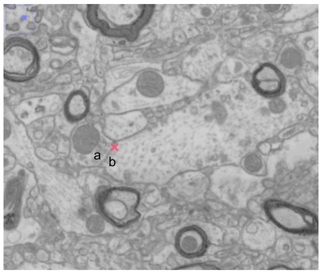	140-180	140-187	21.86
23	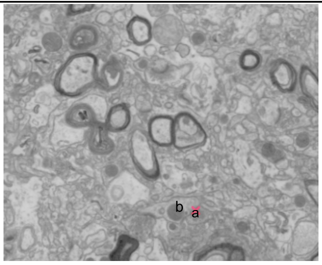	94-134	94-130	18.6
24	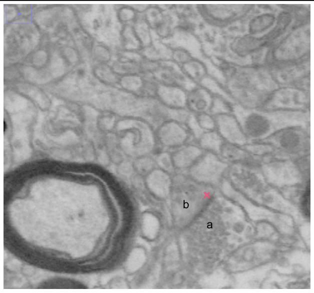	120-133	120-139	8.84
25	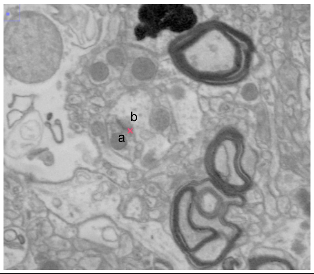	58-100	58-90	19.5
26	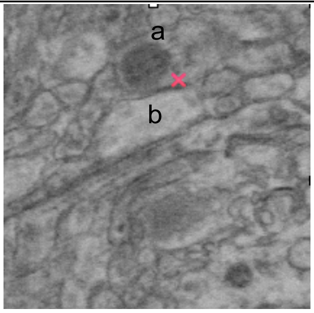	1-57	1-215 (entire sample)	26.52
27	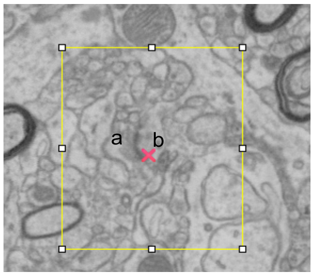	8-88	8-87	37.21

Synapse #	Image	Ability to trace a	Ability to trace b	% Traced through
**28**	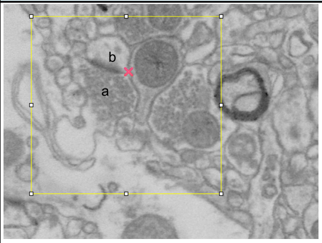	9-95	9-110 (Note: merges into structure at 110, but that structure continues into the end)	40
**29**	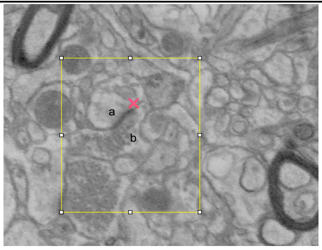	1-60	1-51	23.72
**30**	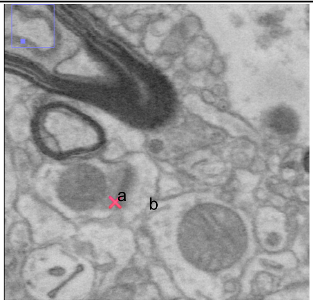	1-128	4-80	35.35
**31**	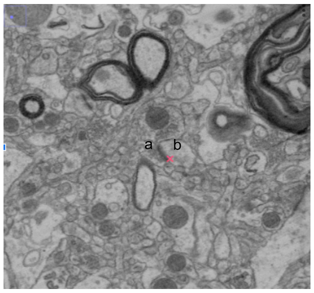	1-28	1-118	13.02
**32**	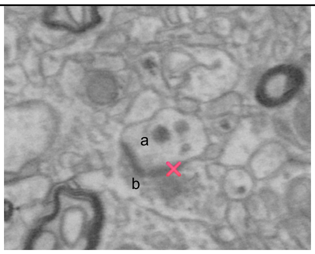	1-73	1-44	20.47
**33**	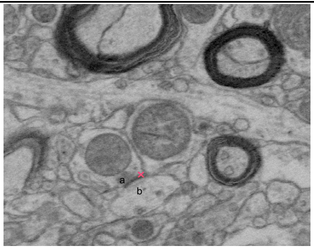	1-55	1-215	25.58

Synapse #	Image	Ability to trace a	Ability to trace b	% Traced through
**34**	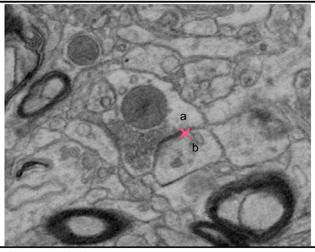	1-23	1-29	10.7
**35**	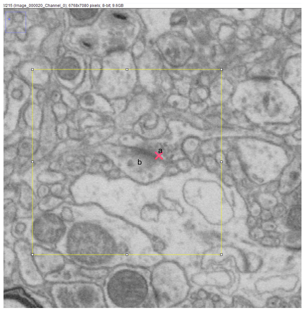	1-21	1-215	9.77
**36**	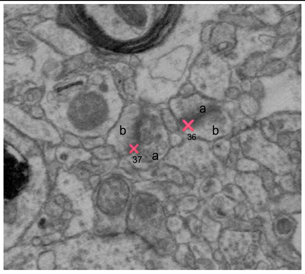	1-108	1-23	10.7
**37**	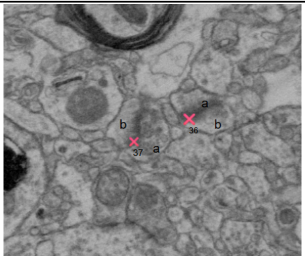	1-56	1-215	26.05
**38**	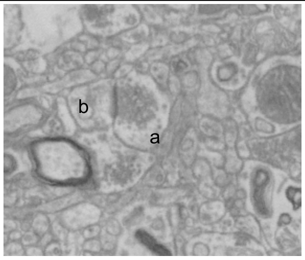	73-135	60-130	28.83
**39**	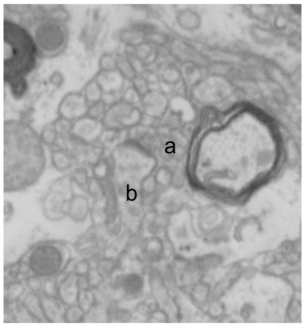	130-215	130-166	16.74

Synapse #	Image	Ability to trace a	Ability to trace b	% Traced through
40	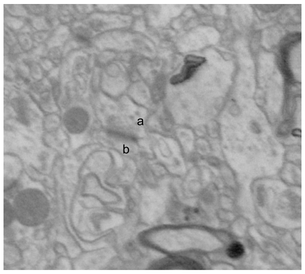	1-165	128-162	15.81
41	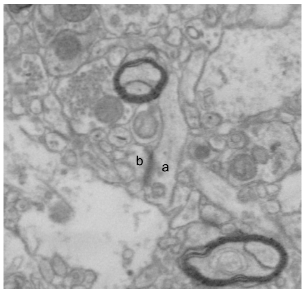	1-215	1-45	20.9
42	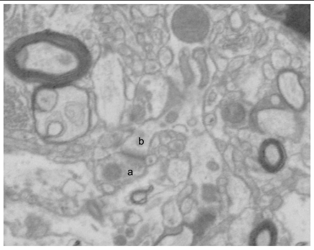	74-215	195-215	9.3
43	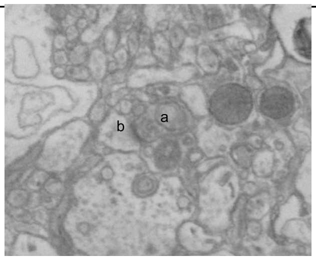	1-215	1-115	53.49
44	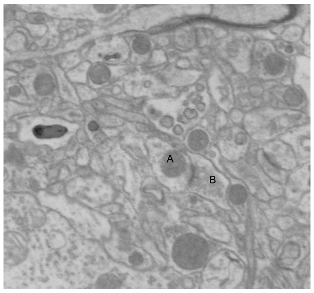	1-215	109-172	29.3
45	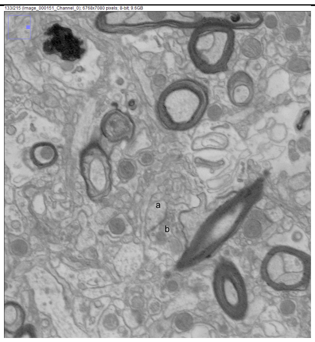	105-200	100-206	48.83

Synapse #	Image	Ability to trace a	Ability to trace b	% Traced through
46	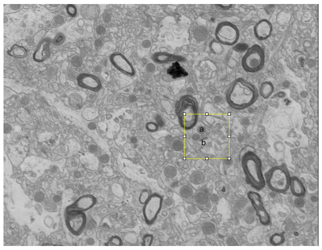	61-151	81-142	28.37
47	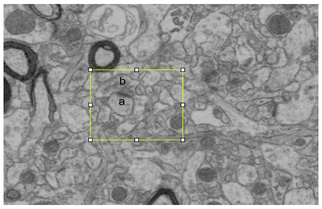	1-50	1-56	23.26
48	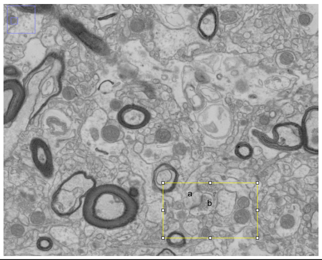	1-65	1-75	34.88
49	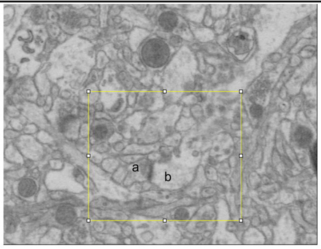	1-113	1-10	4.65
50	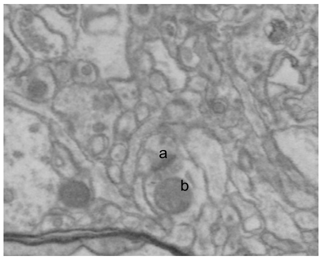	1-47	1-68	21.86
			**Average Percentage Traced**	21.41%

Supplementary Data File 2. Neurite tracing for randomly chosen synapses in volume electron microscopy data.
Volume EM imaging data showing the tracing of the neurites from 50 individual synapses from the frontal cortex of human donor 7 (PMI: 4.25 h). For each synapse, the pre- and postsynaptic neurites were identified at the plane of the synapse and then followed through the z-stack until they could no longer be unambiguously traced to the adjacent section. The “Image” column shows a representative section containing the synapse, with the presynaptic neurite labeled “a” and the postsynaptic neurite labeled “b.” The “Ability to trace a” column reports the range of sections through which the presynaptic neurite could be unambiguously followed, and the “Ability to trace b” column reports the same for the postsynaptic neurite. Cells highlighted in blue indicate neurites that were traceable throughout the entirety of the sample volume (sections 1-215). The “% Traced through” column reports the percentage of the full 215-section sample volume spanned by the traced portion of the neurite pair.
