# Supplementary material for: Expansion microscopy of banked brain tissue
Source: Free Neuropathol. 2026 Jun 29;7:15. doi: 10.17879/freeneuropathology-2026-9593 (PMC13344129; doi:10.17879/freeneuropathology-2026-9593)
Supplement: Supplementary file 3 [file freeneuropathol-07-15-9593-s3.pdf]

Supplementary Data File 3

Pixel Location	Ability to trace a	Ability to trace b	% Traced through	Image
349 at (-6973 1.80e4 1.18e4 1.1834) um	1-26	1-52	10.4	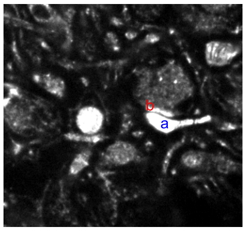
364 at (-7164 1.80e4 1.18e4) um	1-196	35-66	39.1	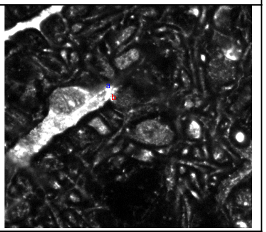
511 at (-7097 1.80e4 1.18e4) um	326-421	302- 386	16.7	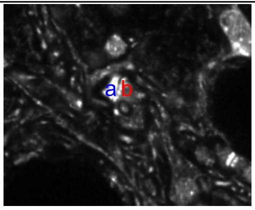
641 at (-7174 1.80e4 1.18e4) um	378-498	369-448	23.95	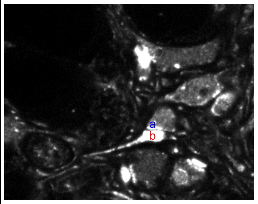
519 at (-7162 1.80e4 1.18e4) um	392-501	380-405	21.8	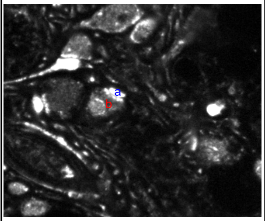
352 at (-6968 1.80e4 1.18e4) um	368-422	368-423	10.9	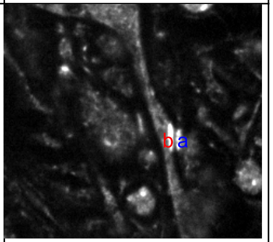
604 at (-7096 1.81e4 1.18e4) um	265-360	297-357	18.96	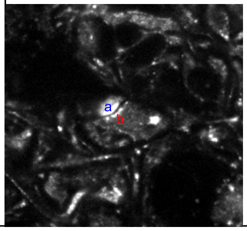

Pixel Location	Ability to trace a	Ability to trace b	% Traced through	Image
266 at (-7045 1.81e4 1.18e4) um	305-350	227-334	21.4	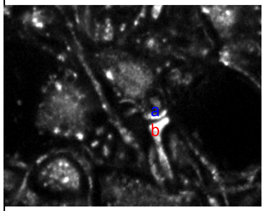
309 at (-6987 1.81e4 1.18e4) um	318-470	410-456	30.3	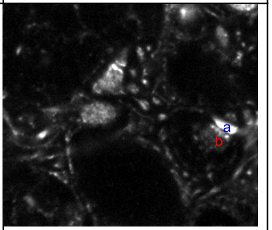
381 at (-7010 1.81e4 1.18e4) um	400-484	400-501	20.2	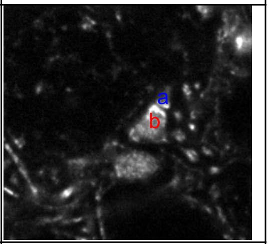
473 at (-7155 1.80e4 1.18e4) um	1-44	1-73	14.6	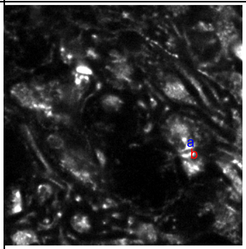
242 at (-7226 1.80e4 1.18e4) um	1-65	1-120	23.95	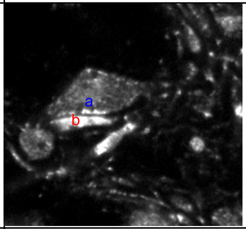
246 at (-7182 1.80e4 1.18e4) um	1-60	1-52	5.9	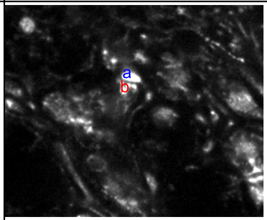
505 at (-7210 1.81e4 1.18e4) um	2-30	1-23	5.59	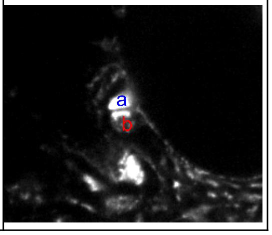

Pixel Location	Ability to trace a	Ability to trace b	% Traced through	Image
586 at (-7052 1.79e4 1.18e4) um	9-64	1-49	10.98	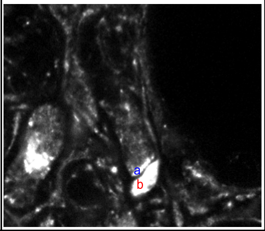
230 at (-7134 1.79e4 1.18e4) um	206-257	187-256	13.77	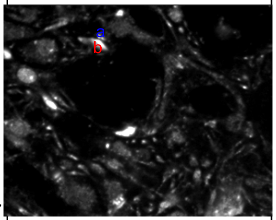
278 at (-7152 1.80e4 1.18e4) um	330-409	332-401	15.77	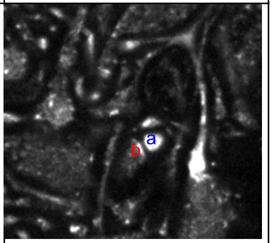
584 at (-7068 1.80e4 1.18e4) um	476-501	462-501	7.78	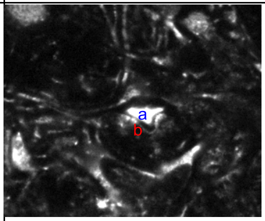
238 at (-7236 1.8034 1.18e4) um	245-307	204-305	20.16	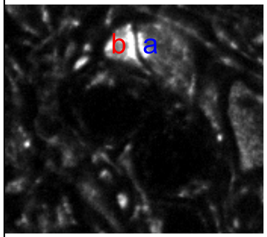
252 at (-7088 1.82e4 1.18e4) um	368-399	340-456	23.15	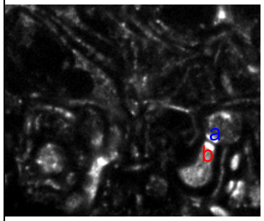
185 at (-6972 1.80e4 1.19e4) um	465-501	461-501	7.98	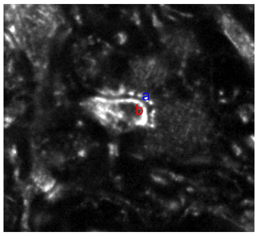

Pixel Location	Ability to trace a	Ability to trace b	% Traced through	Image
257 at (-7102 1.81e4 1.18e4) um	299-336	226-340	22.75	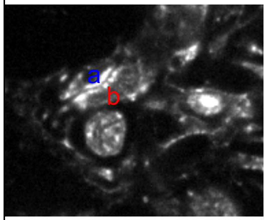
191 at (-7024 1.80e4 1.18e4) um	70-130	84-126	11.98	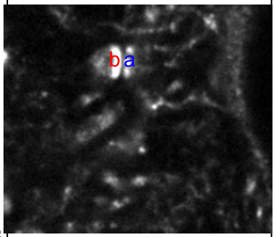
675 at (-7054 1.80e4 1.18e4) um	68-133	68-134	13.17	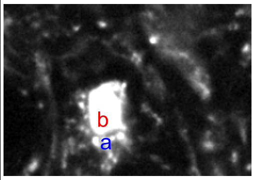
362 at (-7247 1.80e4 1.18e4) um	114-160	114-167	10.58	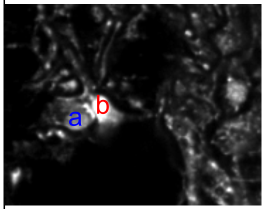
238 at (-7161 1.79e4 1.18e4) um	27-137	81-138	21.96	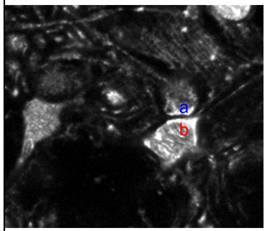
250 at (-7132 1.80e4 1.18e4) um	118-169	115-168	10.58	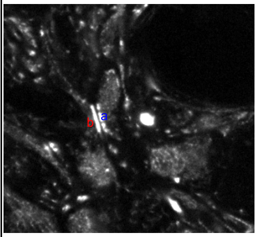
497 at (-7112 1.81e4 1.18e4) um	165-226	170-228	12.2	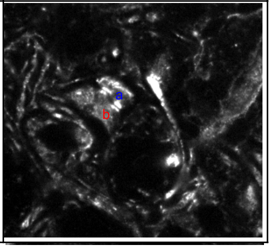

Pixel Location	Ability to trace a	Ability to trace b	% Traced through	Image
353 at (-7235 1.79e4 1.18e4) um	288-329	295-334	8.18	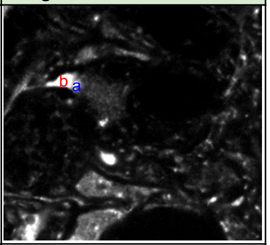
176 at (-7045 at 1.79e4 1.18e4) um	183-245	183-245	12.38	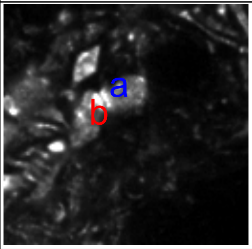
445 at (-6998 1.81e4 1.18e4) um	32-128	30-130	19.96	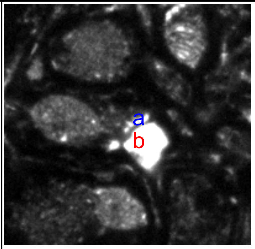
296 at (-7043 1.81e4 1.18e4) um	57-112	57-115	11.58	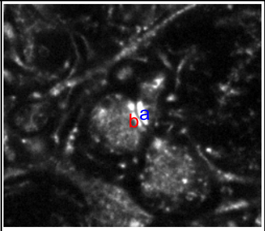
145 at (-7043 1.81e4 1.18e4) um	5-56	1-106	21.26	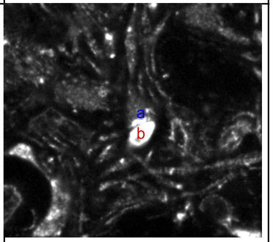
541 at (-7142 1.79e4 1.18e4) um	125-176	120-153	10.18	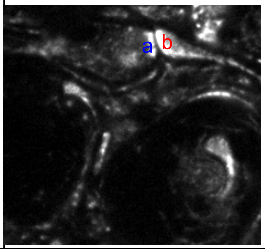
529 at (-7218 1.80e4 1.18e4) um	266-321	258-324	13.17	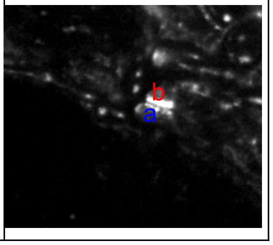

Pixel Location	Ability to trace a	Ability to trace b	% Traced through	Image
653 at (-7006 1.81e4 1.18e4) um	227-381	227-370	30.7	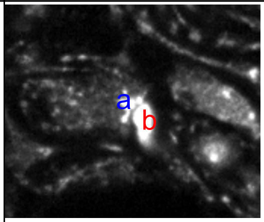
187 at (-7043 1.79e4 1.18e4) um	171-211	171-225	10.78	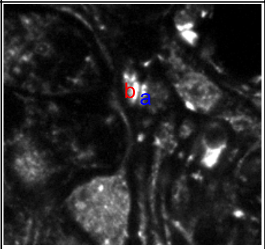
405 at (-7207 1.79e4 1.18e4) um	143-220	148-218	15.37	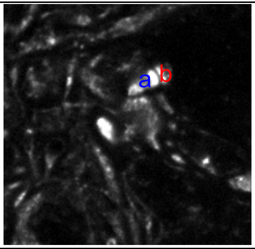
393 at (-7248 1.80e4 1.18e4) um	115-150	117-170	10.58	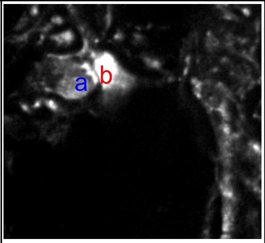
334 at (-7225 1.81e4 1.18e4) um	116-149	127-166	7.78	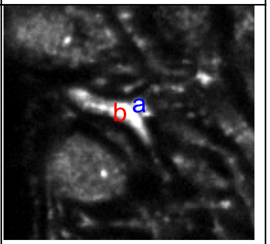
356 at (-7228 1.81e4 1.18e4) um	115-152	119-160	8.18	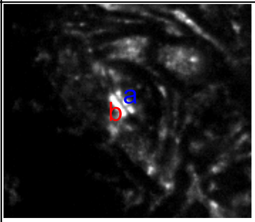
278 at (-7115 1.81e4 1.18e4) um	165-229	166-225	12.77	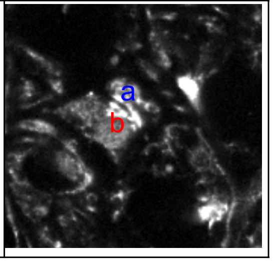

Pixel Location	Ability to trace a	Ability to trace b	% Traced through	Image
430 at (-7153 1.80e4 1.18e4) um	274-321	245-303	9.84	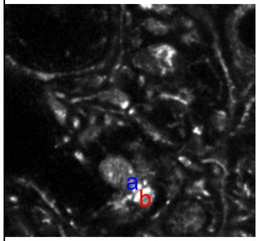
256 at (-7268 1.81e4 1.18e4) um	339-400	349-400	12.18	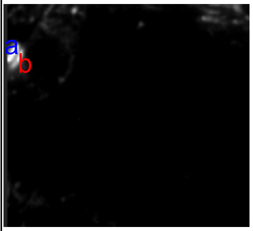
757 at (-7005 1.81e4 1.18e4) um	331-372	331-380	9.78	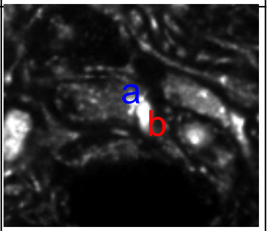
179 at (-6967 1.79e4 1.18e4) um	99-135	105-132	7.19	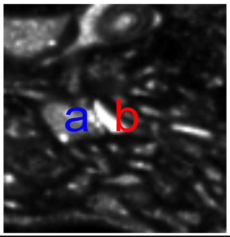
772 at (-6997 1.79e4 1.18e4) um	71-170	88-133	19.76	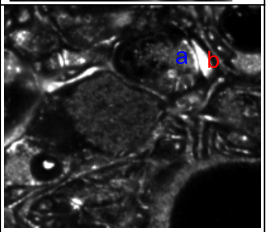
467 at (-7003 at 1.79e4 1.18e4) um	94-117	93-112	4.6	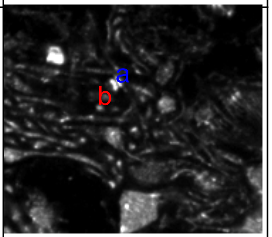
250 at (-6993 at 1.79e4 1.18e4) um	68-129	73-140	13.37	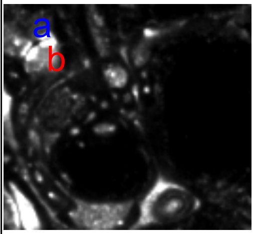

Pixel Location	Ability to trace a	Ability to trace b	% Traced through	Image
229 at (-7037 1.79e4 1.18e4) um	90-139	91-128	9.78	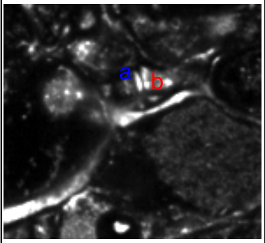
			Average % traced: 14.9186	

Supplementary Data File 3. Neurite tracing for randomly chosen synapses in volume expansion microscopy data.

Expansion microscopy imaging data showing the tracing of the neurites from 50 individual synapses from the frontal cortex of human donor 7 (PMI: 4.25 h). For each synapse, the pre- and postsynaptic neurites were identified at the plane of the synapse and then followed through the z-stack until they could no longer be unambiguously traced to the adjacent section. The “Pixel Location” column gives the section number and (x, y, z) coordinates in microns at which the synapse was identified. The “Image” column shows a representative section containing the synapse, with the presynaptic neurite labeled “a” and the postsynaptic neurite labeled “b.” The “Ability to trace a” column reports the range of sections through which the presynaptic neurite could be unambiguously followed, and the “Ability to trace b” column reports the same for the postsynaptic neurite. The “% Traced through” column reports the percentage of the full 501-section sample volume spanned by the traced portion of the neurite pair.
